# Effects of transcutaneous electrical nerve stimulation based on wrist-ankle acupuncture theory on the skeletal muscle repair and gut microbiota modulation in rats with gastrocnemius muscle injury

**DOI:** 10.3389/fresc.2026.1742110

**Published:** 2026-09-14

**Authors:** Haobin Cheng, Tingting Mai, Kai Chen, Mengcheng Cai, Fanfu Fang

**Affiliations:** 1 College of Basic Medicine, Naval Medical University, Shanghai, China; 2 Department of Rehabilitation, Changhai Hospital of Shanghai, Naval Medical University, Shanghai, China

**Keywords:** fibrosis, gut microbiota, inflammation, muscle repair, WAA-TENS

## Abstract

**Background:**

In recent years, the gut microbiota has attracted considerable attention as a critical regulator of inflammation and metabolism, particularly due to its involvement in muscle repair via the “gut-muscle axis.” This study aims to evaluate the therapeutic efficacy of transcutaneous electrical nerve stimulation based on wrist-ankle acupuncture theory (WAA-TENS) in a rat model of gastrocnemius muscle injury, and subsequently to explore whether its effects are associated with alterations in the gut microbiota.

**Methods:**

Twenty-four 8-week-old rats were randomly allocated to three groups (*n* = 8 per group): a control group (CON), a model group (MOD), and a WAA-TENS group. The CON group received intramuscular saline, whereas gastrocnemius injury induced by complete Freund's adjuvant (CFA) was established in the MOD and WAA-TENS groups; the latter received WAA-TENS. Mechanical pain threshold, local cytokines, and histological outcomes were assessed, and fecal samples were analyzed by 16S rRNA gene sequencing.

**Results:**

Behavioral studies indicated that WAA-TENS significantly increased the pain threshold of model rats (*p* < 0.001). Histological analysis revealed that the degree of inflammatory cell infiltration in the WAA-TENS group was less than that in the MOD group, and the degree of fibrosis was significantly lower than that in the MOD group. Bacterial diversity analysis indicated that the microbiota structure in the MOD group did not differ significantly from that in the CON group, suggesting that CFA-induced local muscle injury alone may be insufficient to induce systemic gut dysbiosis under the present experimental conditions. Notably, WAA-TENS significantly altered the microbiota structure (characterized by a significant decrease in evenness and an increase in the abundance of Lactobacillus), suggesting that WAA-TENS may exert distinct modulatory effects on the intestinal microenvironment independent of the injury-induced response.

**Conclusion:**

WAA-TENS effectively alleviated mechanical allodynia, attenuated local inflammation, and promoted muscle repair in a rat model of CFA-induced gastrocnemius injury. Concurrently, WAA-TENS was associated with significant alterations in gut microbiota composition, including the enrichment of Lactobacillus reuteri. These parallel findings suggest a potential association between WAA-TENS-induced microbiota modulation and its therapeutic effects on skeletal muscle, providing a hypothesis-generating foundation for future mechanistic investigations into the gut-muscle axis. The causal contribution of specific microbial taxa to muscle repair, however, remains to be established.

## 1 Introduction

Muscle injury refers to structural damage, functional impairment, or metabolic dysfunction of muscle tissue, including myofibers, surrounding fascia, tendons, and other associated structures—resulting from trauma, overuse, or pathological conditions (1). As one of the most prevalent musculoskeletal disorders affecting individuals in daily life and occupational settings, muscle injury not only leads to pain and reduced work capacity but also imposes a substantial socioeconomic burden (2). Currently, the gold standard for acute muscle injury in clinical practice is the “RICE” principle (rest, ice, compression, and elevation). However, robust evidence regarding its impact on long-term patient prognosis is lacking. In 2020, the Journal of Sports Medicine proposed a more comprehensive “PEACE” principle (protection, elevation, avoidance of anti-inflammatory drugs, compression, and education), which is applicable to the acute stage of injury (3). Although non-steroidal anti-inflammatory drugs (NSAIDs) are commonly used for analgesia in acute soft tissue injuries, adverse events affecting the skin and gastrointestinal tract still occur (4). Furthermore, there is evidence suggesting that NSAIDs may inhibit muscle repair (3), therefore, it is not recommended to use NSAIDs during the acute phase of muscle injury. However, in clinical practice, completely avoiding the use of NSAIDs may reduce patient satisfaction. Severe pain also limits functional activities, thereby hindering the implementation of early exercise rehabilitation. Therefore, there is an urgent need to find a treatment strategy that effectively alleviates pain while simultaneously promoting muscle repair.

Wrist-Ankle Acupuncture (WAA), a form of superficial needling therapy, has demonstrated unique advantages in pain management (5). However, due to its invasive nature, WAA poses a risk of infection. Furthermore, the therapeutic efficacy of this approach is highly dependent on the practitioner's skill and clinical experience, thereby hindering its standardization and widespread application. Transcutaneous electrical nerve stimulation (TENS) is a non-invasive technique that transmits low-frequency electrical pulses through surface electrode pads to alleviate pain and improve local function (6). Its application is characterized by non-invasiveness and quantifiability, effectively addressing the limitations associated with WAA. We have developed a transcutaneous electrical nerve stimulation device based on wrist-ankle needle theory (WAA-TENS) by combining WAA and TENS, previous studies by our research group have demonstrated that WAA-TENS exerts significant analgesic effects in the management of myofascial pain syndrome, rotator cuff injury, and non-sedative colonoscopy (7–9). Furthermore, WAA-TENS delivers quantifiable stimulation to acupoints, thereby facilitating the standardization of WAA. Nevertheless, its potential role in promoting muscle repair, along with the underlying effects and mechanisms, remains to be systematically elucidated. The aim of this study is to determine whether WAA-TENS can exert dual effects of analgesia and muscle repair, and to elucidate the underlying mechanism of action in order to reveal its scientific significance.

The “gut-muscle axis” represents a conceptual framework for a bidirectional communication network linking the gut and muscle tissues. It refers to the mutual influence and regulation between the gut (especially the gut microbiota and its metabolic activities) and skeletal muscle in terms of physiological functions, metabolic states, and disease occurrence through multiple pathways (10–12). The proposal of this concept has challenged the view that “muscle health is only determined by exercise, nutrition or hormones”. Emerging evidence indicates that WAA and TENS can positively modulate the gut microbiota (13, 14). Therefore, we hypothesize that WAA-TENS alleviates pain and promotes muscle repair through mechanisms potentially involving the gut microbiota. This study aims to investigate the underlying mechanism of WAA-TENS from the perspective of gut microbiota, thereby providing a scientific foundation for its future clinical application and translation.

## 2 Methods

### 2.1 Animal experiment design

Twenty-four healthy male Sprague-Dawley (SD) rats of specific-pathogen-free (SPF) grade, aged 8 weeks and weighing 270–320 g, were obtained from Zhejiang Charles River Laboratory Animal Technology Co., Ltd. and housed at the Experimental Animal Center of Changhai Hospital, Naval Medical University. The rats were maintained under a 12-hour light-dark cycle, at a temperature of 24–25 ℃ and a humidity of 40%–50%, with *ad libitum* access to food and water. The rats were housed individually in separate cages to exclude the influence of coprophagy on the gut microbiota. All animal experimental procedures were reviewed and approved by the Ethics Committee of Changhai Hospital [CHEC(A.E)2025-014]. After one week of acclimatization in the SPF environment, the rats were randomly divided into three groups (*n* = 8 per group) using a random number table method: the control group (CON group), the model group (MOD group), and the WAA-TENS group (15).

### 2.2 Main reagents and instruments

Complete Freund's adjuvant (CFA; Sigma, USA, F5881), tribromoethanol (Aibei, Nanjing, CN, M2820), low-frequency electronic pulse therapy apparatus (Jiajian Medical, CN), Von Frey Hairs mechanical stimulation needles (Yuyan Instruments, CN), Von Frey test cage (Yuyan Instruments, CN), Rat IL-6 ELISA Kit (Servicebio, CN, GER0001-96T), Rat IL-1β ELISA Kit (Servicebio, CN, GER0002-96T), Rat TNF-α ELISA Kit (Servicebio, CN, GER0004-96T), Rat TGF-*β* ELISA Kit (Servicebio, CN, GER0051-96T), BCA Protein Assay Kit (Servicebio, CN, G2026-1000T).

### 2.3 Modeling method

The rats were weighed and anesthetized by intraperitoneal injection of tribromoethanol (1.2 mL/100 g). The hind limbs were shaved, and the rats were positioned in a prone restraint device. Using a 1-mL syringe, 200 µL of CFA was injected into each gastrocnemius muscle to establish a model of muscle inflammatory pain at a depth of approximately 5 mm, with the needle retained for 30 s after injection to minimize leakage. The model was considered successful when gastrocnemius muscle swelling was observed and the plantar mechanical pain threshold was significantly reduced (16).

### 2.4 Intervention methods

In the CON group, an equal volume of normal saline was injected into the gastrocnemius muscle, and a 1-cm-diameter electrode was applied to the lower 1 area without electrical stimulation. (lower 1 area defined in rats as 1 cm proximal to the medial malleolus along the medial border of the Achilles tendon) In the MOD group, CFA was injected, and the electrode was applied in the same manner without electrical stimulation. In the WAA-TENS group, CFA was injected, and the electrode was applied to the lower 1 area to deliver dense-disperse waveform stimulation at 2/100 Hz and 1 mA. All interventions were administered for 20 min once daily for 3 consecutive days.

### 2.5 Pain threshold detection methods

All von Frey assessments were performed by the same trained investigator, who was blinded to group allocation. The paw withdrawal threshold (PWT) was measured by von Frey filaments at 3 h after the last intervention and after the successful modeling. A glass cage of 10 cm × 12 cm × 60 cm was placed on a metal mesh frame 40 cm high, with a metal wire mesh at the bottom of the cage. Each rat was acclimatized in the cage for 20 min until its activity and grooming behavior stopped. The “up-down” method was used, and the labeled forces of the von Frey filaments were 0.4 g, 0.6 g, 1.4 g, 2.0 g, 4.0 g, 6.0 g, 8.0 g, 10.0 g, and 15.0 g (17). The measurement started with the 4.0 g filament, and the mid-plantar region of the hind paw was stimulated. The filament was bent to a “C” or “S” shape and held for 6–8 s. The rat's response was observed and recorded. If there was no obvious response, it was recorded as a negative response (O), and the filament with a larger labeled force was used for stimulation. If the hind paw was rapidly withdrawn, it was recorded as a positive response (X), and the filament with a smaller labeled force was used for stimulation. Each stimulation lasted for several seconds. When an “OX” or “XO” crossover occurred, four more measurements were made to obtain a sequence of “O” and “X” combinations. This sequence was imported into the Up-Down Reader analysis tool to calculate the mechanical pain threshold of the rat (18).

### 2.6 Fecal and gastrocnemius muscle sampling

Following the final behavioral assessment, feces were obtained directly from the rat's anus using sterile forceps. When no feces were present, gentle abdominal massage was applied to stimulate defecation. The excreted feces were stored in sterile EP tubes, flash-frozen in liquid nitrogen, and stored at −80 °C for subsequent analysis. Following anesthesia administration, the skin of the lower leg was surgically incised, the underlying fascia was carefully dissected, and the gastrocnemius muscle was fully exposed for subsequent procedures. A 1 × 1 cm tissue block was excised from the middle of the muscle belly. The left side was fixed in 4% paraformaldehyde solution, and the right side was placed in a cryotube, flash-frozen in liquid nitrogen and then transferred to a −80 °C freezer.

### 2.7 ELISA test

The gastrocnemius muscle tissue was homogenized in ice water to prepare a 10% tissue homogenate. After centrifugation, the supernatant was taken for determination. The expression levels of IL-1β, IL-6, TNF-α and TGF-β in the tissue were determined according to the kit instructions. After the reaction was terminated, the absorbance was measured using a SuPerMax 3,100 multimode microplate reader (Shanghai Flash Spectrum Biotechnology Co., Ltd., Shanghai, China), and the concentration of each analyte was calculated based on the established standard curve.

### 2.8 HE staining

The gastrocnemius samples were fixed in 4% paraformaldehyde for 24 h, embedded in paraffin with the tissue oriented to obtain transverse sections relative to the myofiber long axis, and cut into 3–4 μm sections. The sections were deparaffinized, rehydrated, stained with hematoxylin and eosin (HE), dehydrated, cleared, and mounted (19).

### 2.9 Masson staining

Gastrocnemius muscle tissues were fixed in 4% paraformaldehyde for 24 h, embedded in paraffin, and transversely sectioned at a thickness of 4 μm. After deparaffinization and rehydration, the sections were stained with Weigert's iron hematoxylin and Ponceau-acid fuchsin solution, differentiated with phosphomolybdic acid, and counterstained with aniline blue to visualize collagen fibers. The sections were subsequently dehydrated, cleared, and mounted (20).

### 2.10 Quantitative histology

HE- and Masson-stained sections were digitized using an SQS-120P slide scanning system (Shenzhen Shengqiang Technology Co., Ltd., Shenzhen, China) at a nominal scanning magnification of 20× and a spatial resolution of 0.207 μm/pixel under identical scanning settings. The whole-slide images were examined using ImageScope, and representative images were exported at displayed magnifications of 5× and 20×. Although eight animals were included in each group, histological sections were prepared and retained from six animals per group owing to resource limitations during tissue processing. Thus, six independent animals per group were included in the histological quantification. Five non-overlapping fields were manually sampled from spatially distributed, artifact-free regions containing predominantly transverse myofiber profiles using the same criteria across groups, and the mean of the five fields was used as the animal-level quantitative value. All images were analyzed using custom Python scripts with identical fixed parameters. In HE images, inflammatory infiltration was quantified based on clusters of hematoxylin-positive nuclei; in Masson images, aniline-blue-stained collagen deposition was segmented to determine the fibrotic area. After blank or cavity regions were excluded, inflammatory infiltration and fibrotic area were expressed as percentages of the effective tissue area. The exploratory analysis of myofiber minimum Feret diameter (MFD) was performed using the same Masson-stained transverse sections. Detailed field-selection criteria and image-analysis procedures are provided in Supplementary Material S1 (20, 21).

### 2.11 Intestinal microbiota sequencing

Total DNA of microbial communities was extracted according to the instructions of the E.Z.N.A. soil DNA kit. DNA concentration and purity (A260/A280 ratio) were determined using NanoDrop2000. PCR amplification of the V3-V4 variable region of the 16S rRNA gene was performed using the forward primer 338 (5′-ACTCCTACGGGAGGCAGCAG-3′) and the reverse primer 806 (5′-GGACTACHVGGGTWTCTAAT-3′). PCR products were recovered using 2% agarose gel and purified with the AxyPrep DNA Gel Extraction Kit. The recovered products were quantified using the Quantus Fluorometer. Sequencing was conducted on the Illumina MiSeq PE300/NovaSeq PE250 platform. Sequence processing and microbial community analyses were performed following previously described procedures using mothur and the Majorbio Cloud Platform (22–24).

### 2.12 Statistical analysis

Statistical analyses were performed using GraphPad Prism software (version 9.5). Prior to statistical analysis, normality was assessed using the Shapiro–Wilk test, and homogeneity of variance was evaluated with Levene's test. Multiple-group comparisons were conducted by one-way analysis of variance (ANOVA) followed by Tukey's HSD *post hoc* test, while differences between two independent groups were assessed using the independent samples *t*-test. For microbial community data, between-sample comparisons were carried out with the Wilcoxon rank-sum test. Multiple-group analyses involving biologically independent replicates were performed using the Kruskal–Wallis test, and categorical data were evaluated by the chi-square test. Data are presented as the mean ± standard deviation. Statistical significance was defined as follows: *p < 0.05; **p < 0.01; ***p < 0.001; ****p < 0.0001

## 3 Results

### 3.1 WAA-TENS can increase the mechanical pain threshold of the rat's plantar surface

As shown in Figure 1A, compared with the CON group (15 g), the mechanical pain threshold of rats in the MOD group was significantly reduced to 2.86 g (*p* < 0.0001), indicating the successful establishment of the CFA-induced inflammatory pain model. After WAA-TENS intervention, the mechanical pain threshold recovered to 7.61 g. Although the mechanical pain threshold of the rat's plantar surface did not return to the normal level, it was significantly improved compared with the MOD group (*p* < 0.001), suggesting that WAA-TENS can effectively alleviate CFA-induced mechanical allodynia.

**Figure 1:**
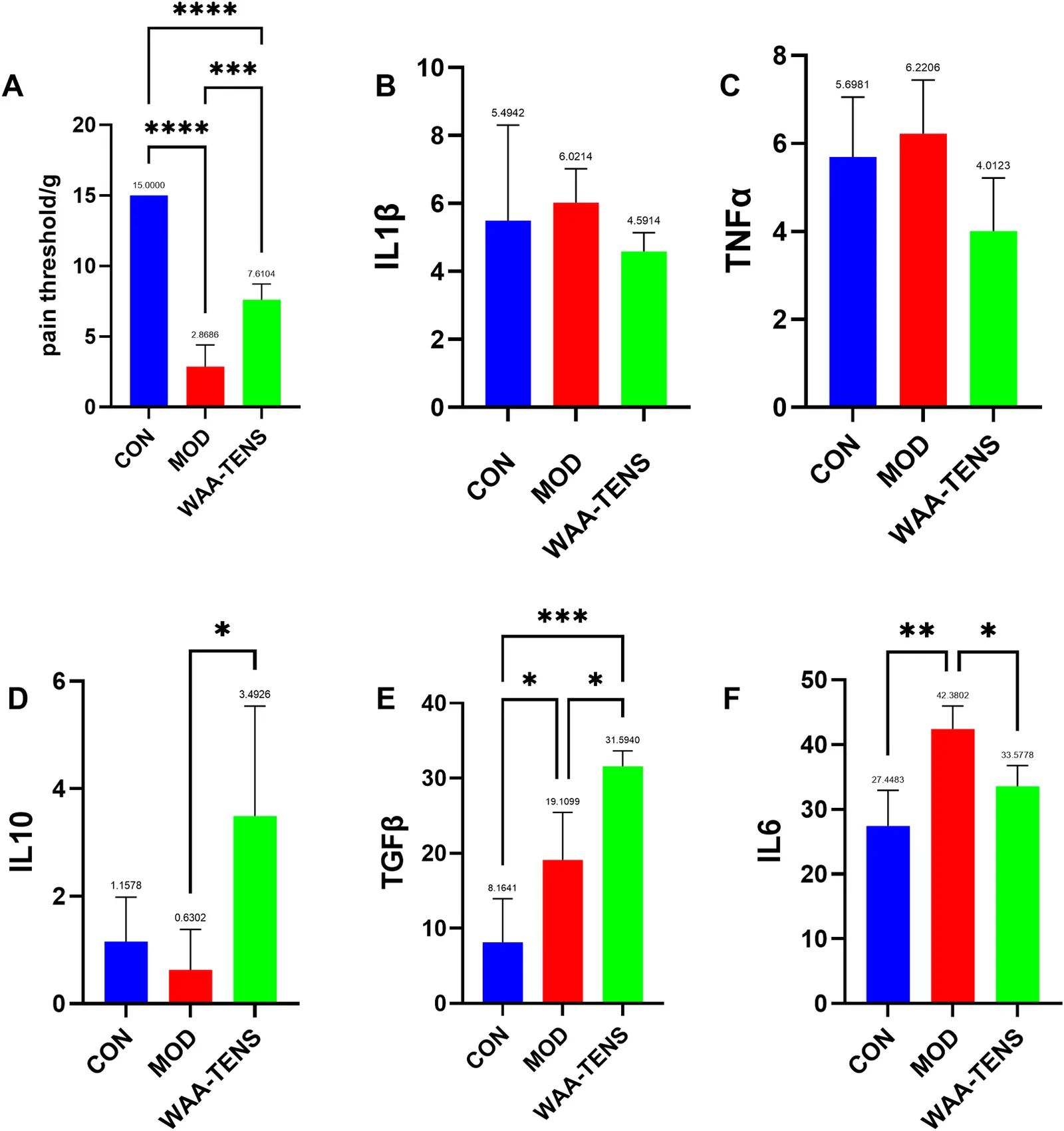
Effects of WAA-TENS on pain threshold and cytokine profiles in a rat model of muscle injury. Statistical comparisons were performed using one-way analysis of variance (ANOVA) followed by Tukey's HSD *post hoc* test. Significant differences are indicated as: *p < 0.05, **p < 0.01, ***p < 0.001, ****p < 0.0001. **(A)** Mechanical pain threshold (unit: g) (vs. CON group: ****p < 0.0001; vs. MOD group: ***p < 0.001). **(B)** Relative expression level of pro-inflammatory cytokine interleukin-1β (IL-1β); **(C)** Relative expression level of pro-inflammatory cytokine tumor necrosis factor-α (TNF-α); **(D)** Relative expression level of anti-inflammatory cytokine interleukin-10 (IL-10) (vs. MOD group: *p* < 0.05); **(E)** Relative expression level of tissue repair-related factor transforming growth factor-β (TGF-β) (vs. CON group: *p* < 0.05; vs. MOD group: *p* < 0.001); **(F)** Relative expression level of pro-inflammatory cytokine interleukin-6 (IL-6) (vs. CON group: *p* < 0.01; vs. MOD group: *p* < 0.05); CON, control group (no muscle injury, no treatment); MOD, muscle injury model group (muscle injury, no treatment); WAA-TENS, muscle injury group treated with WAA combined with TENS.

### 3.2 WAA-TENS can improve the inflammatory factor indicators in rats

The ELISA results are shown in Figures 1B–F. The relative expression levels of pro-inflammatory cytokine IL-6 and tissue repair-related factor TGF-*β* in the MOD group were higher than those in the CON group (*p* < 0.05). The expression of IL-6 in the WAA-TENS group was significantly lower than that in the MOD group, while the expression of TGF-β was significantly higher than that in the MOD group (*p* < 0.05). The expression of anti-inflammatory cytokine IL-10 was down-regulated in the MOD group and increased after WAA-TENS intervention (*p* < 0.05). There was no statistical difference in IL-1β and TNF-α among the three groups (*p* > 0.05). Overall, these results indicate that WAA-TENS was associated with a distinct local cytokine profile characterized by lower IL-6 levels and higher IL-10 and TGF-β levels, suggesting that WAA-TENS may modulate the local inflammatory milieu and potentially create conditions favorable for tissue recovery.

### 3.3 WAA-TENS can promote muscle repair

HE and Masson staining (Figure 2) showed that, in the CON group, the muscle fibers were regularly arranged with clearly defined structures, minimal inflammatory cell infiltration (0.1%), and a small fibrotic area (5.9%). In the MOD group, the muscle fibers were disordered, with markedly increased inflammatory infiltration (29.2%) and an enlarged fibrotic area (34.0%). Compared with the MOD group, the WAA-TENS group exhibited significantly reduced inflammatory infiltration (2.0%, *p* < 0.0001) and a significantly smaller fibrotic area (14.5%, *p* < 0.0001). Nevertheless, the fibrotic area remained higher than that in the CON group (*p* < 0.01). We also performed an exploratory analysis of the myofiber minimum Feret diameter (MFD). Compared with the MOD group, the MFD in the WAA-TENS group showed a numerical shift toward the CON level, although the difference did not reach statistical significance (Supplementary Figure S1). Together with the significant reductions in inflammatory infiltration and fibrotic area, the overall histological findings suggest a certain degree of improvement in the histological condition of injured muscle following WAA-TENS.

**Figure 2:**
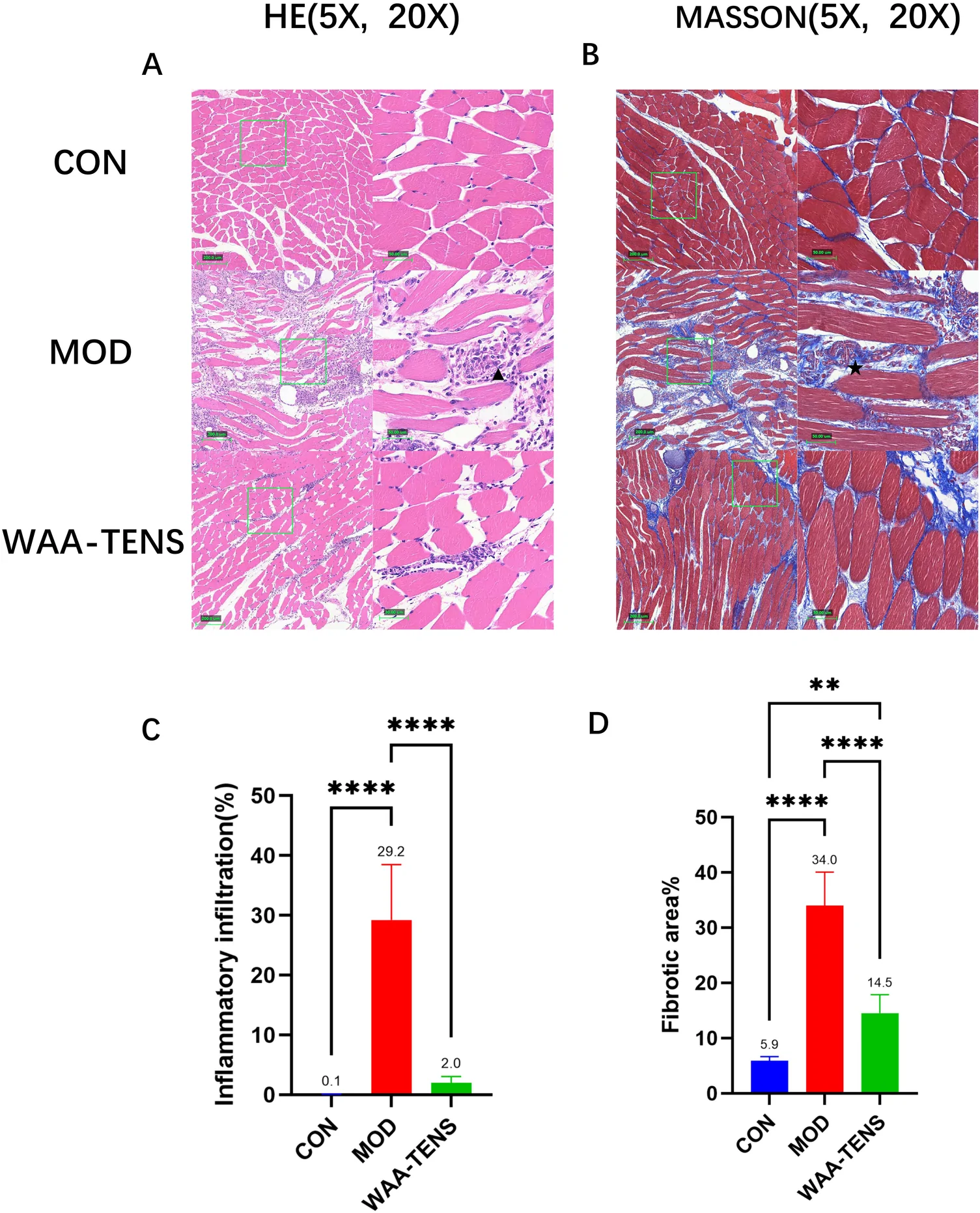
Histopathological changes and quantitative analysis of muscle tissue in a rat model of muscle injury. Statistical comparisons were performed using one-way analysis of variance (ANOVA) followed by Tukey's HSD *post hoc* test. **(A)** Hematoxylin and eosin (HE) staining images (magnification: 5×, 20×) showing muscle tissue architecture (e.g., myofiber integrity and the presence of necrosis) and inflammatory cell infiltration, which were used to evaluate muscle injury severity and the inflammatory response. Inflammatory cells are indicated by solid black triangles (▴). **(B)** Masson staining images (magnification: 5×, 20×) showing collagen fiber deposition (blue-stained regions indicate fibrotic areas), which were used to assess post-injury fibrosis severity. Areas of collagen deposition are indicated by solid black five-pointed stars (★). **(C)** Quantitative analysis of the inflammatory infiltration area percentage (derived from HE staining, reflecting the intensity of the inflammatory response). **(D)** Quantitative analysis of the fibrotic area percentage (derived from Masson staining, reflecting the severity of post-injury fibrosis). Scale bars: 200 μm (5×) and 50 μm (20×).

### 3.4 WAA-TENS can regulate gut microbiota

#### 3.4.1 Inter-group differences in alpha diversity

The *α*-diversity analysis showed no significant differences between the CON and MOD groups for any evaluated index (all *p* > 0.05; Figure 3), indicating no detectable effect of CFA-induced injury on within-sample microbial richness or diversity. Compared with the MOD group, the WAA-TENS group showed significantly lower Sobs, Ace, and Chao indices (*p* < 0.05; Figures 3A–E), and the Chao index was also lower than that in the CON group (*p* < 0.05). Shannon and Simpson did not differ significantly among the three groups (*p* > 0.05; Figures 3B,C), indicating that the reduction in richness was not accompanied by a detectable change in diversity. Coverage was significantly higher in the WAA-TENS group than in the MOD group (*p* < 0.05; Figure 3F). Taken together, these findings suggest that WAA-TENS exerted a measurable influence on the gut microbiota.

**Figure 3:**
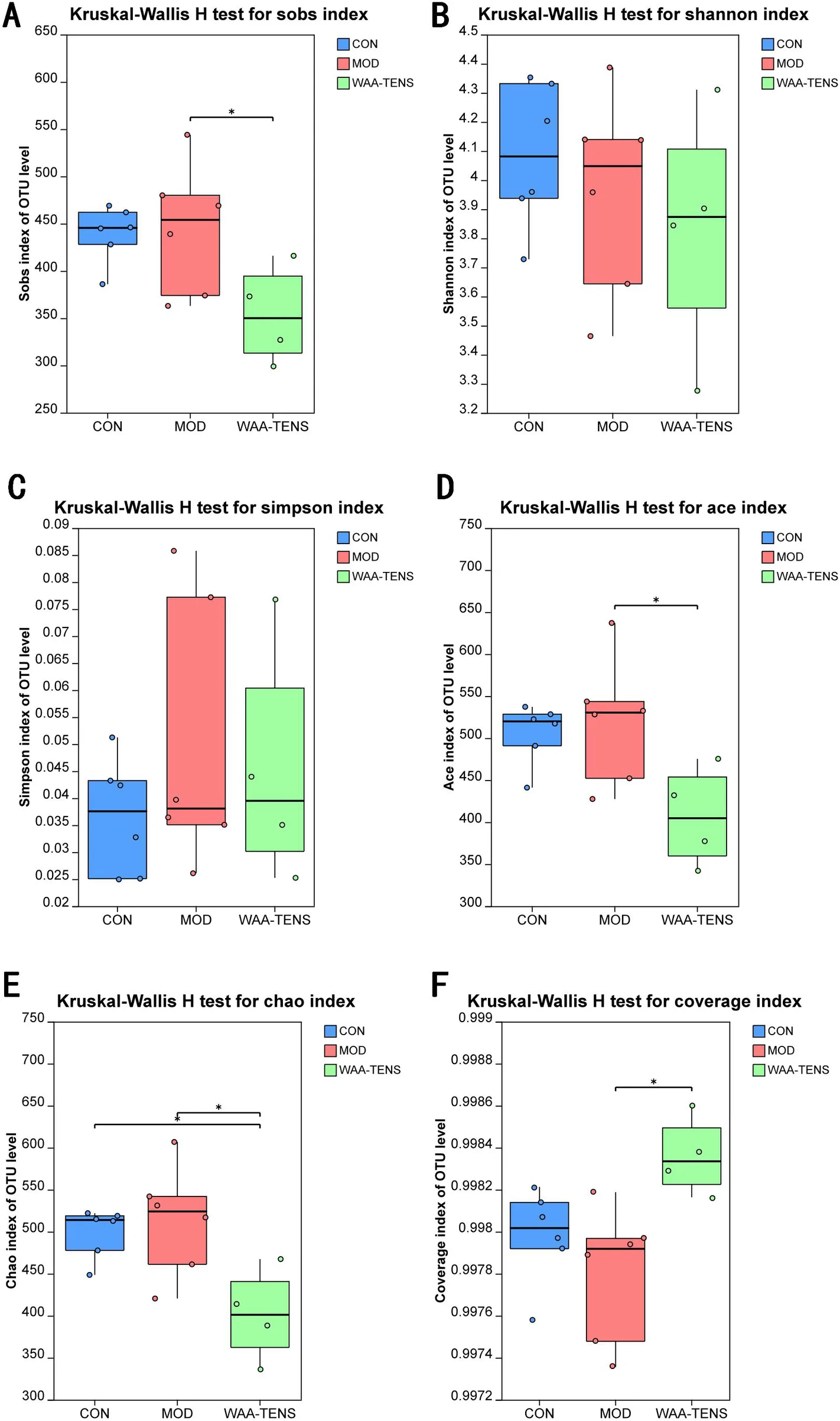
Alpha diversity analysis of Gut Microbiota in rats Among control (CON), muscle injury model (MOD), and WAA-TENS (WAA-TENS) groups. **(A–F)** Box plots showing different alpha diversity indices of gut microbiota in rats from each group: **(A)** Sobs index (reflecting species richness); **(B)** Shannon index (reflecting species diversity); **(C)** Simpson index (reflecting species diversity); **(D)** Ace index (estimator of species richness); **(E)** Chao index (estimator of species richness). **(F)** Coverage index (reflecting sequencing coverage); Statistical analysis was conducted using the Kruskal–Wallis *H*-test. *p* < 0.05 indicates significant differences between groups.

#### 3.4.2 Inter-group differences in beta diversity

The rank-abundance curve (Figure 4A) exhibits a gradual and smooth decline, indicating a relatively even and diverse species distribution, which supports the reliability of the microbial community sampling. Beta diversity, a metric used to assess compositional differences between microbial communities, was analyzed through principal component analysis (PCA), principal coordinates analysis (PCoA), and non-metric multidimensional scaling (NMDS), As shown in Figures 4B–D. The results consistently demonstrate that the microbial community structure of the WAA-TENS group is significantly distinct from those of both the MOD and CON groups, with clearly separated confidence ellipses (*p* < 0.05). Taken together, these results demonstrate distinct differences in intestinal microbial community structure among the CON, MOD, and WAA-TENS groups, indicating a treatment-associated shift in gut microbiota composition following WAA-TENS.

**Figure 4:**
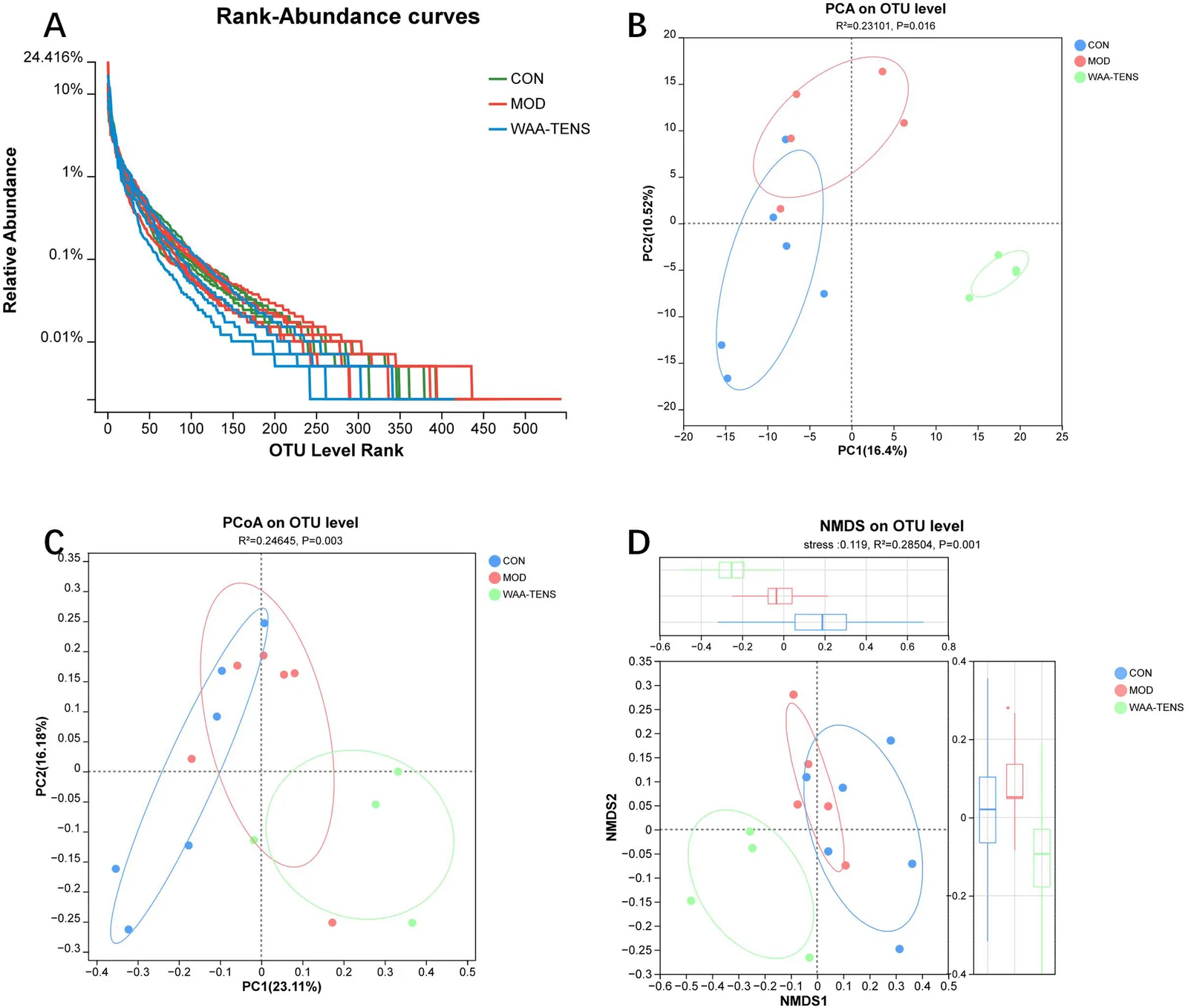
Beta diversity analysis of Gut Microbiota in rats Among control (CON), muscle injury model (MOD), and WAA-TENS groups. **(A)** Rank-Abundance curves illustrating the species richness and evenness of gut microbiota across the CON, MOD, and WAA-TENS groups. **(B)** Principal Component Analysis (PCA) plot at the OTU level, with PERMANOVA results (R^2^ = 0.23101, *p* = 0.016) indicating differences in microbial community structure among groups. **(C)** Principal Coordinates Analysis (PCoA) plot at the OTU level, with PERMANOVA results (R^2^ = 0.24645, *p* = 0.003) reflecting variations in microbial community composition across groups. **(D)** Non-metric Multidimensional Scaling (NMDS) plot at the OTU level, with PERMANOVA results (stress = 0.119, R^2^ = 0.2804, *p* = 0.001) demonstrating dissimilarities in microbial communities among groups.

#### 3.4.3 Analysis of species composition differences at the phylum level

At the phylum level (Figure 5), the Venn diagram (A) reveals that 10 bacterial phyla are shared among the three groups. Bar plots (B) and heatmaps (C) demonstrates intergroup differences in the relative abundances of dominant phyla, including *Bacteroidetes* and *Firmicutes*. Furthermore, phylum Wilcoxon rank-sum tests(D) and linear discriminant analysis effect size (LEfSe) analysis (E; LDA score >4) collectively showed that muscle injury and WAA-TENS treatment were accompanied by changes in the relative abundance of specific taxa, such as *Pseudomonadaceae* and *Acholeplasmataceae* (F). These findings indicate that the improvement in muscle injury outcomes following WAA-TENS was accompanied by distinct changes in the intestinal microbiota. Therefore, a more detailed investigation was conducted at the genus level to identify specific taxonomic drivers of these changes.

**Figure 5:**
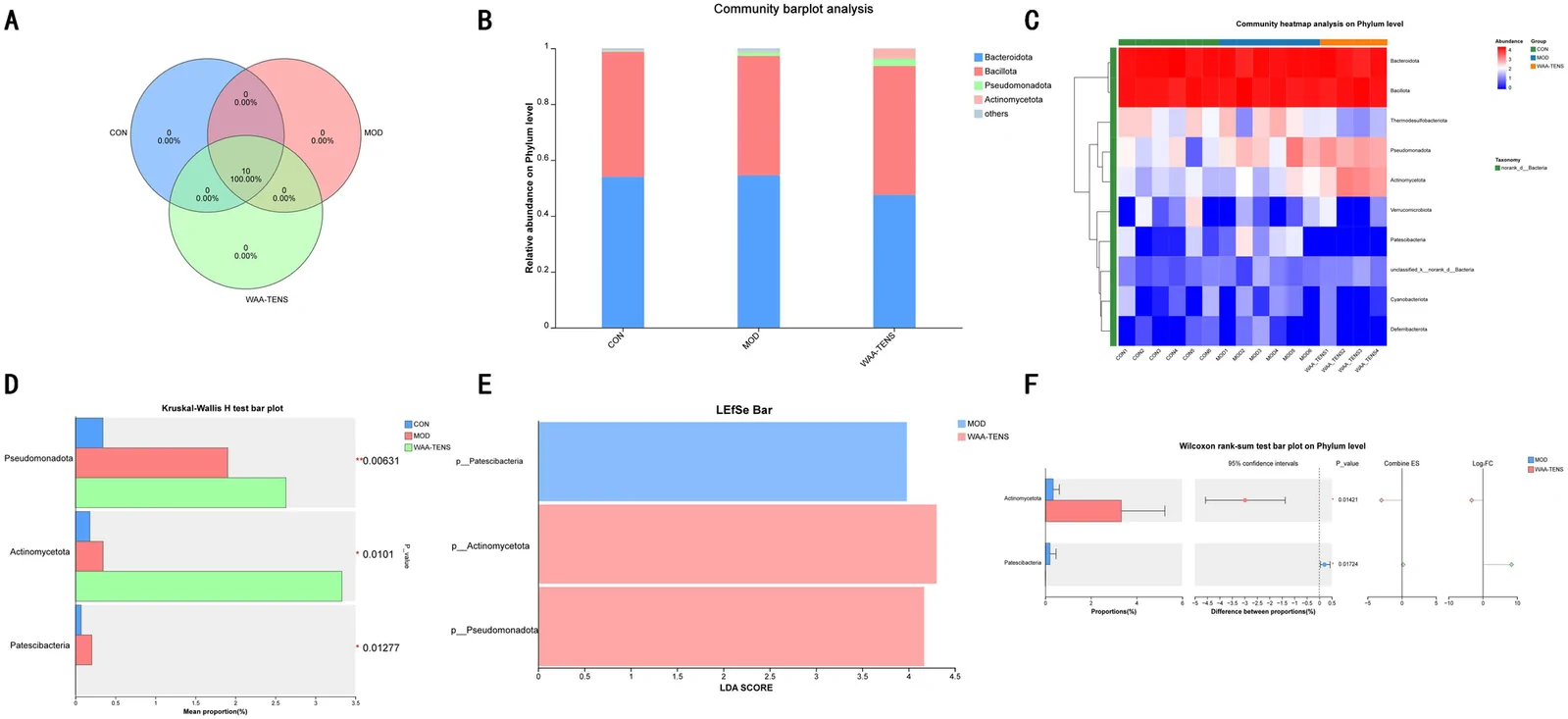
Analysis of gut microbiota community composition at phylum level among CON, MOD, and WAA-TENS groups. **(A)** Venn diagram: illustrates the number of shared and unique phylum across the CON, MOD, and WAA-TENS groups. **(B)** Community bar plot analysis (phylum level): displays the relative abundance of dominant bacterial phyla in each group. **(C)** Community heatmap analysis (phylum level): visualizes the abundance patterns of bacterial phyla, with hierarchical clustering reflecting similarities in microbial community composition across samples. **(D)** Bar plot: shows the mean relative abundance of specific bacterial phylum, with *P*-values indicating statistically significant differences between groups. **(E)** LEfSe bar plot (LDA score): identifies differentially abundant taxa across groups using linear discriminant analysis (LDA) effect size, where an LDA score > 4 indicates taxa enriched in a given group and considered potential microbial biomarkers. **(F)** Wilcoxon rank-sum test bar plot (phylum level): presents proportional differences in bacterial phyla between groups, including 95% confidence intervals and *P*-values for pairwise comparisons.

#### 3.4.4 Analysis of species composition differences at the genus level

At the genus level (Figure 6), the Venn diagram (A) reveals group-shared microbial distributions between three groups. Community bar plots and heatmap analysis at the genus level (B, C) demonstrate intergroup differences in the relative abundances of dominant bacterial genera. Further examination of differentially abundant taxa (D) identified a total of 10 bacterial genera showing statistically significant differences across groups. LEfSe analysis (E; LDA score ≥2) and the Wilcoxon rank-sum test (F) consistently identified genera with differential relative abundance among the groups. These taxa may therefore serve as microbial biomarkers associated with muscle injury and the response to WAA-TENS.

**Figure 6:**
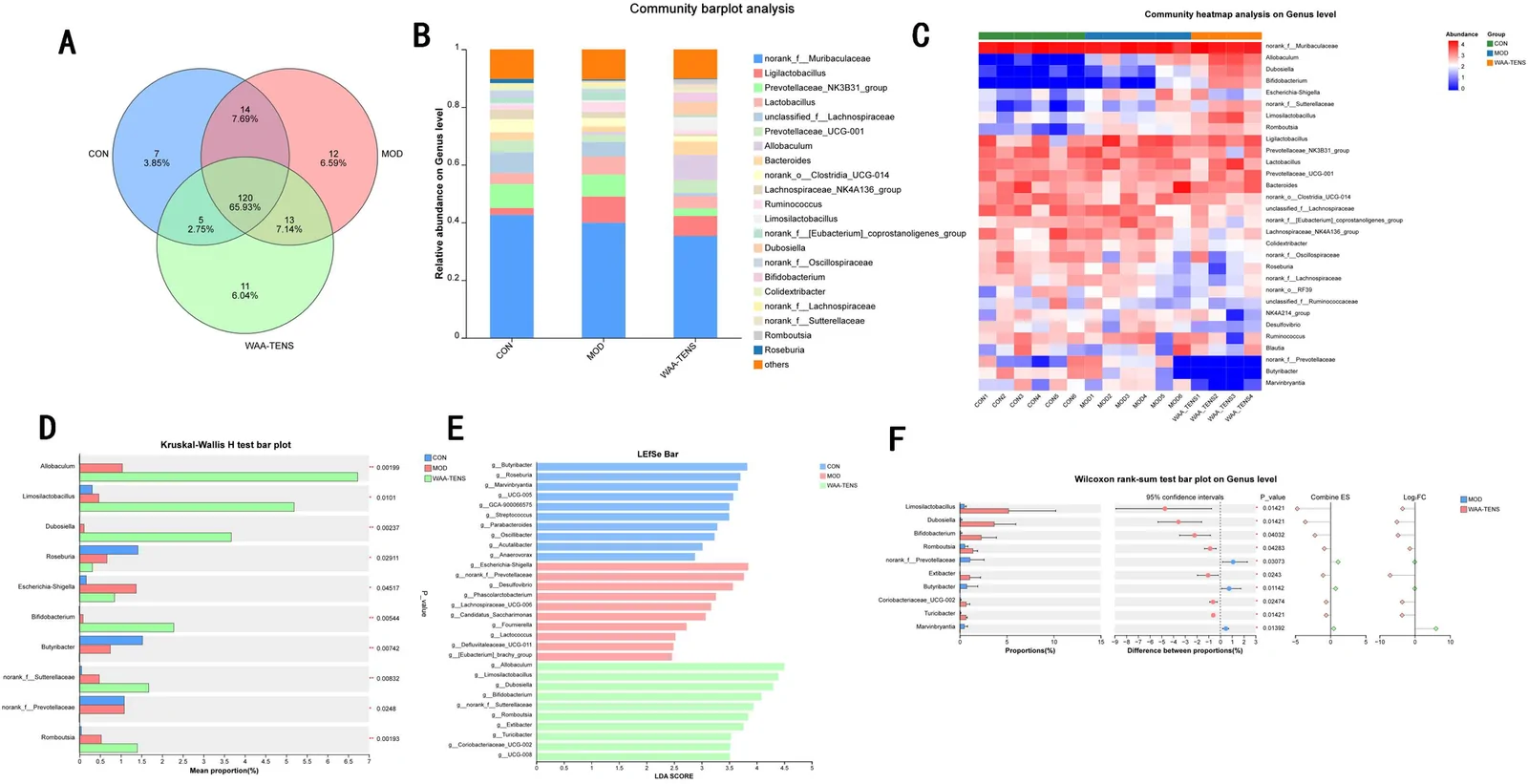
Analysis of gut microbiota community composition at genus level among CON, MOD, and WAA-TENS groups. **(A)** Venn diagram: illustrates the number of shared and unique genus across the CON, MOD, and WAA-TENS groups. **(B)** Community bar plot analysis (genus level): displays the relative abundance of dominant bacterial genera in each group. **(C)** Community heatmap analysis (genus level): visualizes the abundance patterns of bacterial genera, with hierarchical clustering reflecting similarities in microbial community composition across samples. **(D)** Bar plot: shows the mean relative abundance of specific bacterial genera, with *P*-values indicating statistically significant differences between groups. **(E)** LEfSe bar plot (LDA score): identifies differentially abundant taxa across groups using linear discriminant analysis (LDA) effect size, where an LDA score >2 indicates taxa enriched in a given group and considered potential microbial biomarkers. **(F)** Wilcoxon rank-sum test bar plot (genus level): presents proportional differences in bacterial genera between groups, including 95% confidence intervals and *P*-values for pairwise comparisons.

#### 3.4.5 Analysis of species composition differences at the species level

At the species level (Figure 7), it revealed intergroup differences in the relative abundance of dominant bacterial species such as *Lactobacillus reuteri* and *Bifidobacterium animalis*. Further analysis demonstrated that *Lactobacillus reuteri* was significantly differentially abundant between the MOD and WAA-TENS groups, displayed the highest LDA score (4.5). The increased relative abundance of *Lactobacillus reuteri* in the WAA-TENS group was observed alongside improvements in the mechanical pain threshold and histological outcomes, highlighting this species as a candidate taxon for further investigation in relation to the therapeutic response to WAA-TENS.

**Figure 7:**
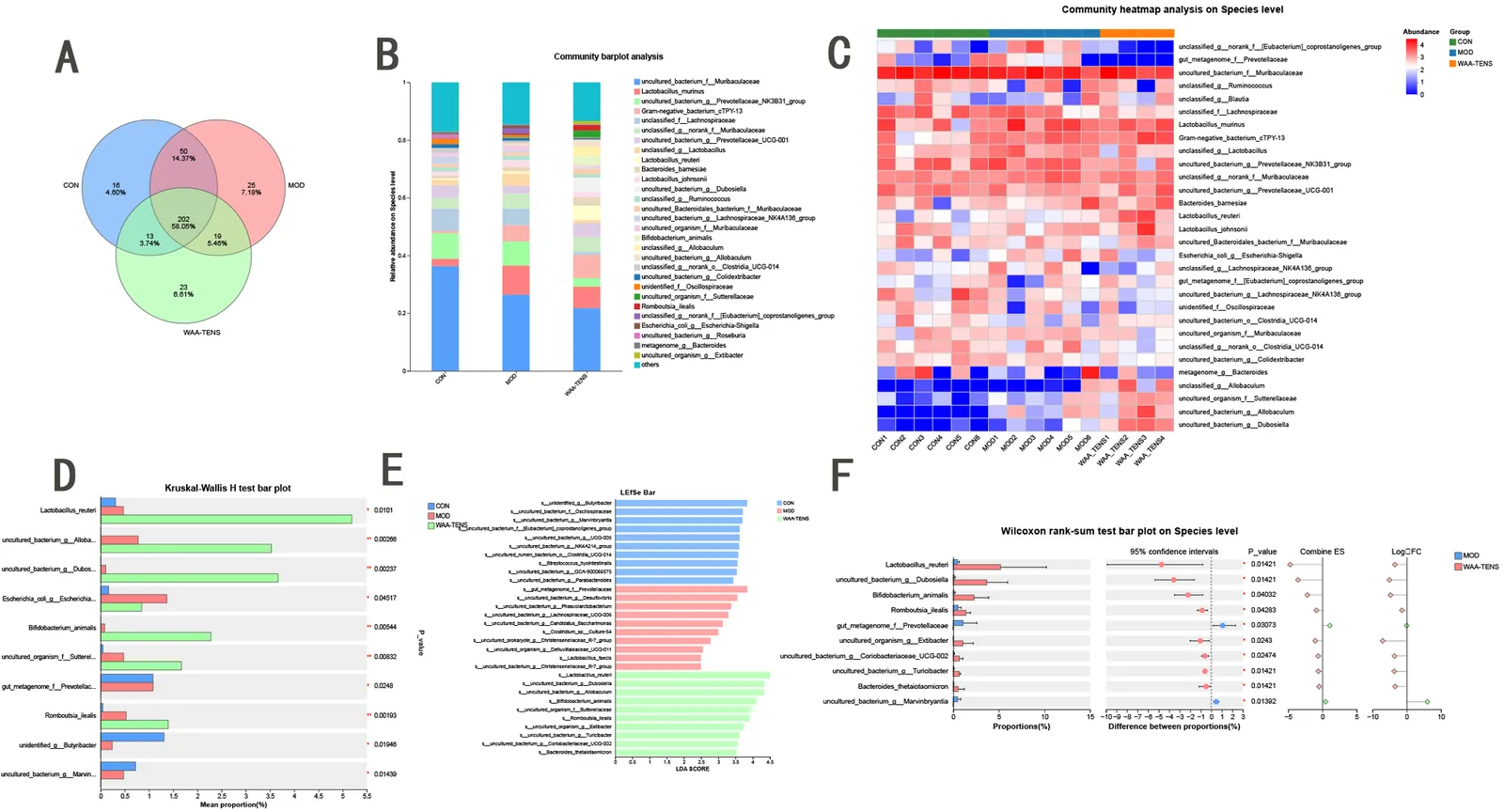
Analysis of gut microbiota community composition at species level among CON, MOD, and WAA-TENS groups. **(A)** Venn diagram: illustrates the number of shared and unique species across the CON, MOD, and WAA-TENS groups. **(B)** Community bar plot analysis (species level): displays the relative abundance of dominant bacterial species in each group. **(C)** Community heatmap analysis (species level): visualizes the abundance patterns of bacterial species, with hierarchical clustering reflecting similarities in microbial community composition across samples. **(D)** Bar plot: shows the mean relative abundance of specific bacterial species, with *P*-values indicating statistically significant differences between groups. **(E)** LEfSe bar plot (LDA score): identifies differentially abundant taxa across groups using linear discriminant analysis (LDA) effect size, where an LDA score > 2 indicates taxa enriched in a given group and considered potential microbial biomarkers. **(F)** Wilcoxon rank-sum test bar plot (species level): presents proportional differences in bacterial species between groups, including 95% confidence intervals and *P*-values for pairwise comparisons.

## 4 Discussion

This study provided convergent evidence from behavioral, biochemical, and histological analyses demonstrating that WAA-TENS exerts significant analgesic and muscle repair-promoting effects in a rat model of CFA-induced gastrocnemius injury. Specifically, WAA-TENS significantly increased the mechanical pain threshold, downregulated the pro-inflammatory cytokine IL-6, upregulated the anti-inflammatory cytokine IL-10 and the tissue repair-related factor TGF-β, and reduced inflammatory infiltration and fibrotic area. In parallel, 16S rRNA sequencing revealed that WAA-TENS intervention was associated with significant shifts in gut microbiota composition, particularly the enrichment of Lactobacillus reuteri. While these dual observations are temporally correlated, the present study design does not permit definitive causal attribution of the therapeutic effects to microbiota modulation. Rather, we interpret the microbiota findings as an exploratory observation that generates a testable hypothesis: WAA-TENS may engage the gut-muscle axis as one component of a multifactorial therapeutic mechanism, warranting future investigations incorporating causal validation approaches such as fecal microbiota transplantation or germ-free models.

The traditional “gate control theory” proposes that TENS activates large-diameter Aβ low-threshold mechanoreceptors, inhibiting the upward transmission of pain signals (transmitted through A*δ* and C fibers) in the spinal dorsal horn, which may be the peripheral basis for the rapid increase in pain threshold in this study. The latest research by the Fudan University team further revealed that TENS can activate the endogenous opioid system through the dorsal root ganglion Aβ low-threshold mechanoreceptors-dorsal column nucleus glutamatergic neurons-ventral posterolateral nucleus of the thalamus glutamatergic neurons-somatosensory hindlimb cortex pathway and recruit the periaqueductal gray/rostral ventromedial medulla descending analgesic network to produce a prolonged analgesic effect (25). The significant improvement in pain threshold in the WAA-TENS group in this study is presumed to be the result of the synergistic effect of “peripheral gate control blockage” and “central opioid regulation”.

After muscle injury occurs, repair proceeds through five interrelated, time-dependent stages: necrosis, inflammation, regeneration, maturation, and functional recovery (26). The abnormal elevation of IL-6, IL-1β and TNF-α plays a key role in initiating the inflammatory cascade and aggravating tissue damage (27–29). Although some studies have shown that it may promote the enrichment of muscle satellite cells and thus contribute in the early stage of regeneration (30–32), excessive inflammatory responses can instead inhibit muscle repair and functional recovery (33). It can even lead to remote organ damage and sepsis (34). In the present study, WAA-TENS significantly downregulated IL-6 and upregulated IL-10, an anti-inflammatory cytokine known to suppress neutrophil rolling and migration (35). These changes support a regulatory effect of WAA-TENS on the local inflammatory microenvironment. It is noteworthy that IL-1β and TNF-α did not differ significantly among the three groups. This may be attributable to several factors, including the selected sampling time point, which may not coincide with the peak expression window of these particular cytokines in the CFA-induced local inflammation model; the relatively localized nature of CFA-induced muscle injury compared to systemic insults; or insufficient statistical power given the sample size. Nevertheless, the significant reduction in IL-6 coupled with the elevation of IL-10 provides consistent evidence that WAA-TENS shifted the local cytokine milieu toward an anti-inflammatory phenotype, thereby alleviating inflammatory pain and creating favorable conditions for tissue repair.

The Masson staining results showed significant differences in fibrotic area among the three groups: 5.9% in the CON group, 34.0% in the MOD group, and 14.5% in the WAA-TENS group. The marked increase in the MOD group was consistent with pathological collagen deposition after acute muscle injury, as the local inflammation triggered by injury can continuously activate fibroblasts and lead to excessive and disorganized collagen deposition (36). Although collagen deposition contributes to filling tissue defects caused by myofiber rupture, persistent inflammation may interfere with the formation of functional repair tissue and consequently hinder muscle recovery. In the WAA-TENS group, the fibrotic area decreased to 14.5%, although it remained higher than that in the CON group. Compared with the MOD group, the WAA-TENS group also showed a significant reduction in inflammatory cell infiltration. Together, these quantitative findings indicate that WAA-TENS attenuated excessive fibrosis and improved the histological condition of the injured muscle. Notably, the increase in tissue TGF-β in the WAA-TENS group was not accompanied by a corresponding increase in fibrotic area at the selected sampling time. This lack of parallel change indicates that tissue TGF-β concentration and accumulated collagen represent distinct endpoints and should not be interpreted as equivalent measures of fibrosis. Therefore, the increased TGF-β level does not provide evidence of aggravated fibrosis, although the present study cannot establish how TGF-β contributed to the observed histological changes. During the physiological repair of acute muscle injury, an appropriate degree of collagen deposition contributes to the regeneration of muscle fibers (36) and provides extracellular matrix support for damaged muscle tissue (37). Taken together, the significant reductions in inflammatory infiltration and fibrotic area suggest a histological pattern consistent with an improved repair process following WAA-TENS treatment. These changes were accompanied by an increased mechanical pain threshold, although the present study did not directly assess whether the histological and behavioral improvements were mechanistically linked.

The gut microbiota-inflammation regulation-muscle repair axis theory posits that the gut microbiota modulates host nutrient metabolism and immune responses, thereby influencing skeletal and muscular health (38, 39). In the present study, 16S rRNA sequencing revealed that WAA-TENS intervention was associated with significant shifts in gut microbiota composition, most prominently the enrichment of *Lactobacillus reuteri* (LEfSe LDA score = 4.5; *p* < 0.05). Notably, the MOD group did not exhibit significant microbiota divergence from the CON group, indicating that CFA-induced local muscle injury alone was insufficient to induce detectable gut dysbiosis under the current experimental conditions. This pattern suggests that the observed microbial alterations in the WAA-TENS group may represent a treatment-specific response rather than a secondary consequence of muscle repair. *Lactobacillus reuteri* has been reported to possess immunomodulatory properties, potentially mediated through the production of short-chain fatty acids (SCFAs) and maintenance of intestinal barrier integrity (40–43). However, in the absence of metabolomic profiling, functional assays, or causal manipulation (e.g., fecal microbiota transplantation or mono-colonization studies), the present data do not permit the conclusion that *L. reuteri* enrichment directly mediates the observed muscle repair effects. An alternative and equally plausible interpretation is that WAA-TENS may modulate gut microbiota composition through neuro-gut axis pathways (e.g., vagal afferent signaling or altered gastrointestinal motility), and that these microbial shifts serve as biomarkers of the systemic physiological response to treatment, rather than causal agents of muscle repair. Therefore, we cautiously interpret the *L. reuteri* finding as a hypothesis-generating observation that warrants targeted mechanistic follow-up.

## 5 Limitations

Several limitations of this study should be acknowledged. First, the study was conducted using a rodent model, and significant interspecies differences in physiology, gut microbiota composition, and muscle injury pathophysiology necessitate cautious extrapolation to human clinical settings. Second, the primary objective of the gut microbiota analysis was hypothesis generation rather than mechanistic validation. While a significant association was observed between WAA-TENS treatment, microbiota composition shifts, and improved muscle repair outcomes, the observational nature of the current design precludes causal inference. Definitive establishment of a causal role for specific microbial taxa would require experimental approaches such as fecal microbiota transplantation (FMT), mono-association studies, or antibiotic depletion followed by selective recolonization, which were beyond the scope of the present investigation. Third, the molecular mechanisms bridging gut microbiota dynamics and muscle regeneration remain incompletely elucidated in this study. The identification of microbial-derived metabolites (e.g., SCFAs, tryptophan metabolites) and the host signaling pathways engaged in this putative regulatory network were not assessed, limiting mechanistic depth. Fourth, neither spontaneous cage activity nor food intake was systematically recorded. Because analgesia may increase movement and feeding, both variables could influence gut microbiota composition and muscle histology and therefore represent potential confounders.

## 6 Conclusion

The present study established, for the first time, a parallel observation that WAA-TENS concurrently exerts analgesic and muscle repair-promoting effects and induces significant alterations in gut microbiota composition in a rat model of CFA-induced muscle injury. These findings provide preliminary experimental support for the “gut-muscle axis” as a relevant framework in the context of electroacupuncture-based neuromodulation, while acknowledging that the causal directionality of the microbiota-muscle relationship remains unresolved. The therapeutic effects of WAA-TENS on muscle repair are likely multifactorial, encompassing established peripheral and central analgesic mechanisms as well as potential contributions from gut microbiota modulation that warrant dedicated mechanistic investigation. From a translational perspective, these results offer preclinical evidence supporting the continued development of WAA-TENS as a non-pharmacological, non-invasive intervention for muscle injury, and generate a testable hypothesis that gut microbial ecology may constitute one modifiable target underlying its systemic effects. These strategies may offer a new avenue to address current challenges in muscle injury management, including high risks of drug-related adverse effects and substantial inter-individual variability in treatment response.

## Data Availability

The original contributions presented in the study are included in the article/Supplementary Material, further inquiries can be directed to the corresponding author/s.

## Supplementary material

SUPPLEMENTARY FIGURE S1. Exploratory analysis of myofiber minimum Feret diameter in gastrocnemius muscle. Minimum Feret diameter (MFD) was quantified using Masson-stained transverse gastrocnemius muscle sections. Bars represent the mean ± standard deviation (*n* = 6 animals per group), and the values above the bars indicate the group means. Statistical comparisons were performed using one-way analysis of variance followed by Tukey's HSD post hoc test. The MFD was numerically higher in the WAA-TENS group than in the MOD group and shifted toward the CON level, although the difference did not reach statistical significance (*p* > 0.05). CON, control group; MOD, muscle injury model group; WAA-TENS, muscle injury group treated with wrist-ankle acupuncture-based transcutaneous electrical nerve stimulation.

## References

[1] Edouard P Reurink G Mackey AL Lieber RL Pizzari T Järvinen TAH. Traumatic muscle injury. Nat Rev Dis Primers. (2023) 9:56. 10.1038/s41572-023-00469-8 37857686

[2] Aguilar-García D Fernández-Sarmiento JA Del Mar Granados Machuca M Rodríguez JM Rascón PM Calvo RN. Histological and biochemical evaluation of plasma rich in growth factors treatment for grade II muscle injuries in sheep. BMC Vet Res. (2022) 18:400. 10.1186/s12917-022-03491-2 PMC9652891 36371198

[3] Dubois B Esculier J-F. Soft-tissue injuries simply need PEACE and LOVE. Br J Sports Med. (2020) 54:72–3. 10.1136/bjsports-2019-101253 31377722

[4] Derry S Moore RA Gaskell H McIntyre M Wiffen PJ. Topical NSAIDs for acute musculoskeletal pain in adults. Cochrane Database Syst Rev. (2015) 2015:CD007402. 10.1002/14651858.CD007402.pub3 26068955 PMC6426435

[5] Xu N Liu L-L Rong W. Wrist-ankle acupuncture as additional therapy for postoperative multimodal analgesia in orthopedic surgery: systematic review and meta-analysis. Pain Med. (2022) 23:1654–69. 10.1093/pm/pnac065 35482527

[6] Johnson MI Paley CA Jones G Mulvey MR Wittkopf PG. Efficacy and safety of transcutaneous electrical nerve stimulation (TENS) for acute and chronic pain in adults: a systematic review and meta-analysis of 381 studies (the meta-TENS study). BMJ Open. (2022) 12:e051073. 10.1136/bmjopen-2021-051073 35144946 PMC8845179

[7] Song W Wang X Zhou J Shi P Gu W Fang F. Rehabilitation of an analgesic bracelet based on wrist-ankle acupuncture in patients with rotator cuff injury: a randomized trial. Pain Res Manag. (2021) 2021:1–8. 10.1155/2021/9946548 PMC8292086 34336070

[8] Du J Shi P Fang F Yu H. Cerebral cortical hemodynamic metrics to aid in assessing pain levels? A pilot study of functional near-infrared spectroscopy. Front Neurosci. (2023) 17:1136820. 10.3389/fnins.2023.1136820 37008231 PMC10050350

[9] Huang X Wang H Shi L Xu L Lv C Song W. Effect of transcutaneous electrical nerve stimulation based on wrist-ankle acupuncture theory for pain relief during colonoscopy without anesthesia: a randomized controlled trial. Endoscopy. (2025) 57:158–65. 10.1055/a-2373-0513 39053503

[10] Lozupone CA Stombaugh JI Gordon JI Jansson JK Knight R. Diversity, stability and resilience of the human gut microbiota. Nature. (2012) 489:220–30. 10.1038/nature11550 22972295 PMC3577372

[11] Yin Y Guo Q Zhou X Duan Y Yang Y Gong S. Role of brain-gut-muscle axis in human health and energy homeostasis. Front Nutr. (2022) 9:947033. 10.3389/fnut.2022.947033 36276808 PMC9582522

[12] Calabrò S Kankowski S Cescon M Gambarotta G Raimondo S Haastert-Talini K. Impact of gut Microbiota on the peripheral nervous system in physiological, regenerative and pathological conditions. Int J Mol Sci. (2023) 24:8061. 10.3390/ijms24098061 PMC10179357 37175764

[13] Guo P Zhang XN Jin XY Xia WJ Zhou L Sheng WS. Clinical efficacy of transcutaneous electrical nerve stimulation (TENS) in pediatric functional constipation: impact on immunological indicators and gut microbiota. J Immunol Res. (2025) 2025:8888730. 10.1155/jimr/8888730 40027592 PMC11870762

[14] Miao M Ma T Chen R Geng K Shen Z Sun Y. TENS Improves CFL injury rat and regulates the intestinal microbiota. PLoS One. (2025) 20:e0319592. 10.1371/journal.pone.0319592 40179112 PMC11967936

[15] Mai T Cai M Tian Y Jiang L Fang F Gu W. Effects and mechanisms of wrist-ankle acupuncture on inflammatory pain in the rat gastrocnemius muscle. J Pain Res. (2026) 19:1–14. 10.2147/JPR.S605568 PMC13265405 42305388

[16] Shi J- Cao W- Zhang X-N Wan H-Y Su Y-S Qu Z-Y. Local analgesia of electroacupuncture is mediated by the recruitment of neutrophils and released *β*-endorphins. Pain. (2023) 164:1965–75. 10.1097/j.pain.0000000000002892 37027145 PMC10436362

[17] Chaplan SR Bach FW Pogrel JW Chung JM Yaksh TL. Quantitative assessment of tactile allodynia in the rat paw. J Neurosci Methods. (1994) 53:55–63. 10.1016/0165-0270(94)90144-9 7990513

[18] Gonzalez-Cano R Boivin B Bullock D Cornelissen L Andrews N Costigan M. Up-down reader: an open source program for efficiently processing 50% von frey thresholds. Front Pharmacol. (2018) 9:433. 10.3389/fphar.2018.00433 29765323 PMC5938897

[19] Cardiff RD Miller CH Munn RJ. Manual hematoxylin and eosin staining of mouse tissue sections. Cold Spring Harb Protoc. (2014) 2014:655–8. 10.1101/pdb.prot073411 24890205

[20] Zhao Y Chang R Zeng C. Protocol for cryoinjury model in neonatal mice for heart regeneration and repair research. STAR Protoc. (2021) 2:100623. 10.1016/j.xpro.2021.100623 34223197 PMC8243160

[21] Dubuisson N Versele R Planchon C Selvais CM Noel L Abou-Samra M. Histological methods to assess skeletal muscle degeneration and regeneration in duchenne muscular dystrophy. Int J Mol Sci. (2022) 23:16080. 10.3390/ijms232416080 36555721 PMC9786356

[22] Schloss PD Westcott SL Ryabin T Hall JR Hartmann M Hollister EB. Introducing mothur: open-source, platform-independent, community-supported software for describing and comparing microbial communities. Appl Environ Microbiol. (2009) 75:7537–41. 10.1128/AEM.01541-09 19801464 PMC2786419

[23] Ren Y Yu G Shi C Liu L Guo Q Han C. Majorbio cloud: a one-stop, comprehensive bioinformatic platform for multiomics analyses. iMeta. (2022) 1:1:e12. 10.1002/imt2.12 PMC10989754 38868573

[24] Wu Q Wang J Tu C Chen P Deng Y Yu L. Gut microbiota of patients insusceptible to olanzapine-induced fatty liver disease relieves hepatic steatosis in rats. Am J Physiol Gastrointest Liver Physiol. (2025) 328:G110–24. 10.1152/ajpgi.00167.2024 39679941

[25] Liu S Long S-S Li F Yang H Pu S Du D. Neural basis of transcutaneous electrical nerve stimulation for neuropathic pain relief. Neuron. (2025) 113:3616–3631.e6. 10.1016/j.neuron.2025.08.010 40925372

[26] Forcina L Cosentino M Musarò A. Mechanisms regulating muscle regeneration: insights into the interrelated and time-dependent phases of tissue healing. Cells. (2020) 9:1297. 10.3390/cells9051297 32456017 PMC7290814

[27] Frangogiannis NG Lindsey ML Michael LH Youker KA Bressler RB Mendoza LH. Resident cardiac mast cells degranulate and release preformed TNF-alpha, initiating the cytokine cascade in experimental canine myocardial ischemia/reperfusion. Circulation. (1998) 98:699–710. 10.1161/01.cir.98.7.699 9715863

[28] Wynn TA Chawla A Pollard JW. Macrophage biology in development, homeostasis and disease. Nature. (2013) 496:445–55. 10.1038/nature12034 23619691 PMC3725458

[29] He Z Ma C Yu T Song J Leng J Gu X. Activation mechanisms and multifaceted effects of mast cells in ischemia reperfusion injury. Exp Cell Res. (2019) 376:227–35. 10.1016/j.yexcr.2019.01.022 30716302

[30] Yang W Hu P. Skeletal muscle regeneration is modulated by inflammation. J Orthop Translat. (2018) 13:25–32. 10.1016/j.jot.2018.01.002 29662788 PMC5892385

[31] Al-Zaeed N Budai Z Szondy Z Sarang Z. TAM Kinase signaling is indispensable for proper skeletal muscle regeneration in mice. Cell Death Dis. (2021) 12:611. 10.1038/s41419-021-03892-5 34120143 PMC8197762

[32] Panci G Chazaud B. Inflammation during post-injury skeletal muscle regeneration. Semin Cell Dev Biol. (2021) 119:32–8. 10.1016/j.semcdb.2021.05.031 34140216

[33] Tu H Li Y-L. Inflammation balance in skeletal muscle damage and repair. Front Immunol. (2023) 14:1133355. 10.3389/fimmu.2023.1133355 36776867 PMC9909416

[34] Mas-Celis F Olea-López J Parroquin-Maldonado JA. Sepsis in trauma: a deadly complication. Arch Med Res. (2021) 52:808–16. 10.1016/j.arcmed.2021.10.007 34706851

[35] Ajuebor MN Das AM Virág L Flower RJ Szabó C Perretti M. Role of resident peritoneal macrophages and mast cells in chemokine production and neutrophil migration in acute inflammation: evidence for an inhibitory loop involving endogenous IL-10. J Immunol. (1999) 162:1685–91. 10.4049/jimmunol.162.3.1685 9973430

[36] Mahdy MAA. Skeletal muscle fibrosis: an overview. Cell Tissue Res. (2019) 375:575–88. 10.1007/s00441-018-2955-2 30421315

[37] Kim J Lee J. Role of transforming growth factor-*β* in muscle damage and regeneration: focused on eccentric muscle contraction. J Exerc Rehabil. (2017) 13:621–6. 10.12965/jer.1735072.536 29326892 PMC5747195

[38] Wang Y Li Y Bo L Zhou E Chen Y Naranmandakh S. Progress of linking gut microbiota and musculoskeletal health: casualty, mechanisms, and translational values. Gut Microbes. (2023) 15:2263207. 10.1080/19490976.2023.2263207 37800576 PMC10561578

[39] Zhang Y-W Wu Y Liu X-F Chen X Su J-C. Targeting the gut microbiota-related metabolites for osteoporosis: the inextricable connection of gut-bone axis. Ageing Res Rev. (2024) 94:102196. 10.1016/j.arr.2024.102196 38218463

[40] Citi S. Intestinal barriers protect against disease. Science. (2018) 359:1097–8. 10.1126/science.aat0835 29590026

[41] Wu H Xie S Miao J Li Y Wang Z Wang M. Lactobacillus reuteri maintains intestinal epithelial regeneration and repairs damaged intestinal mucosa. Gut Microbes. (2020) 11:997–1014. 10.1080/19490976.2020.1734423 32138622 PMC7524370

[42] Xi X Ye Q Li X Lu X Fan D Xia Y. Xiong fu powder regulates the intestinal microenvironment to protect bones against destruction in collagen-induced arthritis rat models. Front Cell Infect Microbiol. (2022) 12:854940. 10.3389/fcimb.2022.854940 35846762 PMC9285403

[43] Di Porzio A Barrella V Cigliano L Mauriello G Troise AD Scaloni A. Diet-induced impairment of skeletal muscle and adipose tissue metabolic homeostasis and its prevention by probiotic administration. Pflugers Arch. (2025) 477:223–39. 10.1007/s00424-024-03041-9 39537965

