# Supplementary material for: Effects of transcutaneous electrical nerve stimulation based on wrist-ankle acupuncture theory on the skeletal muscle repair and gut microbiota modulation in rats with gastrocnemius muscle injury
Source: Front Rehabil Sci. 2026 Sep 14;7:1742110. doi: 10.3389/fresc.2026.1742110 (PMC13617035; doi:10.3389/fresc.2026.1742110)
Supplement: Supplementary file 1 [file Supplementaryfile1.zip › Supplementary_Material_1/01_Documentation/Supplementary_Material_1_Histology_Methods.docx]

**Supplementary Methods and Data Index**

*Quantitative analysis of H&E- and Masson-stained gastrocnemius sections*

# **1. Image sampling and general workflow**

H&E- and Masson-stained sections from six independent animals per group were included in the histological analysis. Although eight animals were included in each experimental group, histological sections were prepared and retained from six animals per group owing to resource limitations during tissue processing. Five non-overlapping fields were analyzed per animal, and the mean of the five field percentages was used as the animal-level result for inflammatory infiltration and fibrotic area. Images were analyzed with custom Python scripts using one fixed parameter set for every group. Blank or cavity regions were excluded from the denominator, and each area-based endpoint was expressed as target area divided by effective tissue area × 100%.

# **2. Selection of fields for histological quantification**

Each whole-slide image was first examined at low magnification using ImageScope. Regions affected by major technical artifacts, including tissue folds, tears, out-of-focus imaging, blank section margins, or markedly uneven staining, were excluded. Because intramuscular CFA administration produced spatially heterogeneous tissue injury, fields were not selected exclusively from either the most severely affected or the least affected regions. Instead, five non-overlapping fields were sampled from spatially separated locations covering the analyzable central and peripheral portions of each section. Eligible fields were required to contain predominantly transverse or near-transverse myofiber profiles, particularly for MFD analysis. The same technical and spatial criteria were applied to all groups. Field selection was completed before quantitative segmentation and was independent of the inflammatory-infiltration, fibrotic-area, or MFD values subsequently generated by the Python scripts. The scripts were applied to the exported fields and did not automatically select regions from the whole-slide images. The five measurements were averaged to obtain a single animal-level value, and animals, rather than individual fields, were treated as the independent units in the statistical analysis.

# **3. H&E inflammatory-infiltration quantification**

The hematoxylin channel was isolated by HED color deconvolution and normalized to 8-bit grayscale. Nuclei were segmented with Otsu thresholding. Dense nuclear clusters were merged by morphological closing, internal holes were filled, and isolated nuclei were removed by morphological opening. At a 2000-pixel image width, the effective closing and opening kernels were 33 × 33 and 13 × 13 pixels, respectively, and connected regions of at least 5120 pixels² were retained as inflammatory-infiltration regions. Parameters were scaled proportionally for the single 2500 × 2500-pixel field. Near-white cavity regions were excluded before calculating the inflammatory-infiltration area percentage.

# **4. Masson fibrotic-area quantification**

Masson images were converted from RGB to HSV color space. Aniline-blue collagen was segmented using hue 95–135 on the OpenCV 0–179 scale, saturation > 35, and value > 60. A 3 × 3-pixel morphological opening removed isolated noise. Near-white cavity regions were excluded, and the segmented blue collagen area was expressed as a percentage of effective tissue area. This percentage is reported in the manuscript and figures as fibrotic area.

# **5. Exploratory myofiber minimum Feret diameter analysis**

Minimum Feret diameter (MFD) was measured from the same 90 full-resolution Masson fields. Images were analyzed at a twofold downsample. The source-image calibration was 0.207217 µm/pixel, corresponding to 0.414435 µm per analysis pixel after downsampling. Red-stained myofiber profiles were segmented using R > 85, R − G > 28, R − B > 30, and G < 190. A 3-pixel minimum filter at the analysis resolution reduced narrow bridges between adjacent profiles. Border-touching objects were excluded. Accepted profiles were required to have an area of 400–10,000 µm², MFD of 15–100 µm, solidity ≥ 0.80, and aspect ratio ≤ 2.5. MFD was calculated as the minimum distance between parallel supporting lines of the convex hull.

For each animal, accepted fibers from the five fields were pooled and the median MFD was used as the independent animal-level value (n = 6 per group).

# **6. Supplementary figure legend**

Supplementary Figure S1. Exploratory analysis of gastrocnemius myofiber minimum Feret diameter. Minimum Feret diameter (MFD) was quantified from Masson-stained muscle sections. Bars represent the mean ± SD (n = 6 animals per group), and the values above the bars indicate the group means. Statistical comparisons were performed using one-way analysis of variance followed by Tukey's HSD post hoc test. The MFD was numerically higher in the WAA-TENS group than in the MOD group and shifted toward the CON level, although the difference did not reach statistical significance (p > 0.05). CON, control group; MOD, muscle injury model group; WAA-TENS, muscle injury group treated with wrist-ankle acupuncture combined with transcutaneous electrical nerve stimulation.
