# Supplementary material for: Effects of transcutaneous electrical nerve stimulation based on wrist-ankle acupuncture theory on the skeletal muscle repair and gut microbiota modulation in rats with gastrocnemius muscle injury
Source: Front Rehabil Sci. 2026 Sep 14;7:1742110. doi: 10.3389/fresc.2026.1742110 (PMC13617035; doi:10.3389/fresc.2026.1742110)
Supplement: Supplementary file 1 [file Supplementaryfile1.zip › Supplementary_Material_1/02_Result_Figures/Histology_Quantification_Result_Charts(1).pdf]

H&E inflammatory-infiltration analysis - CON

Thirty fields (five fields per animal; six animals per group); labels show the quantified area percentage.

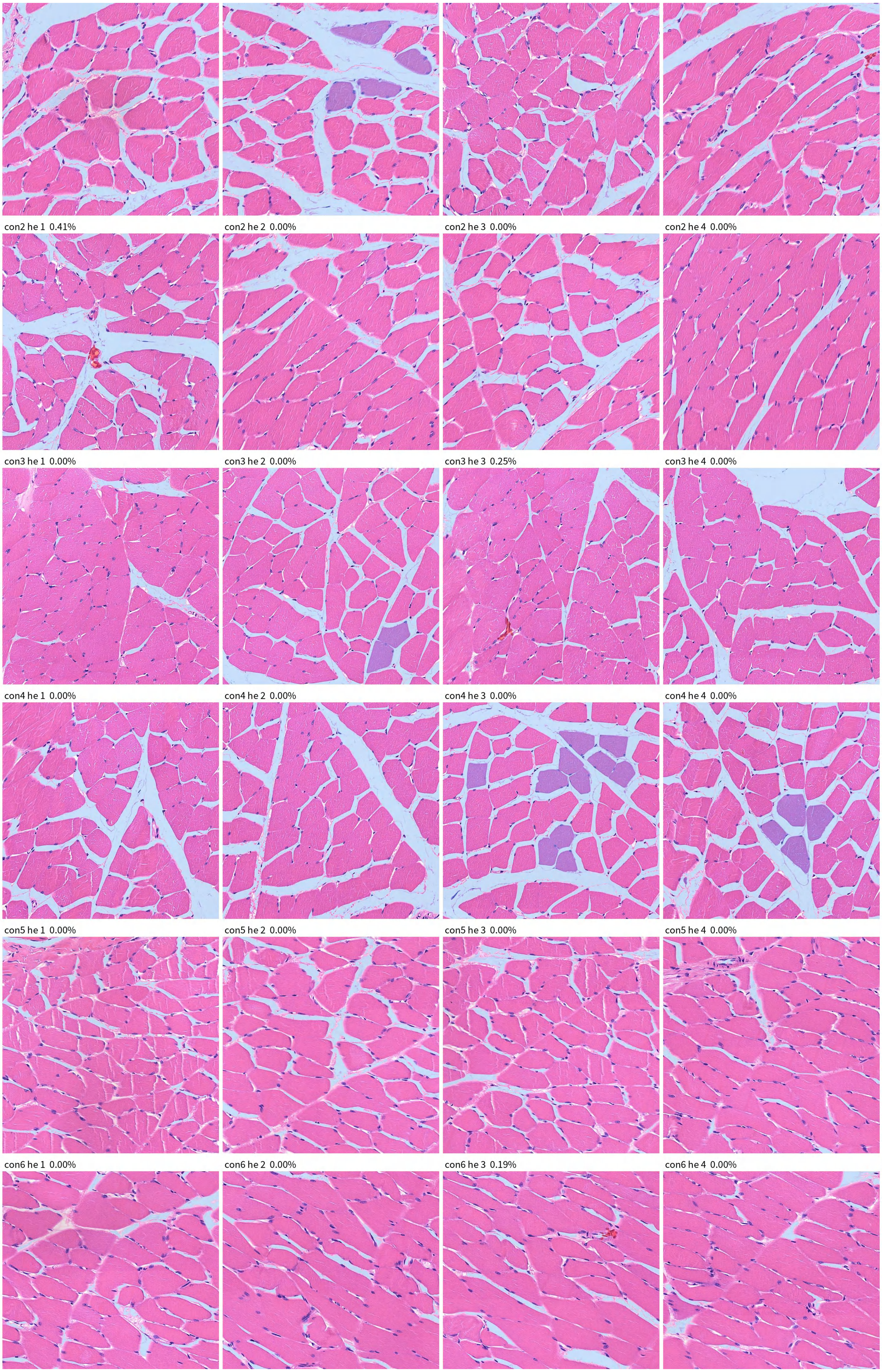

The same fixed analysis parameters were applied to all groups.

H&E inflammatory-infiltration analysis - WAA-TENS

Thirty fields (five fields per animal; six animals per group); labels show the quantified area percentage.

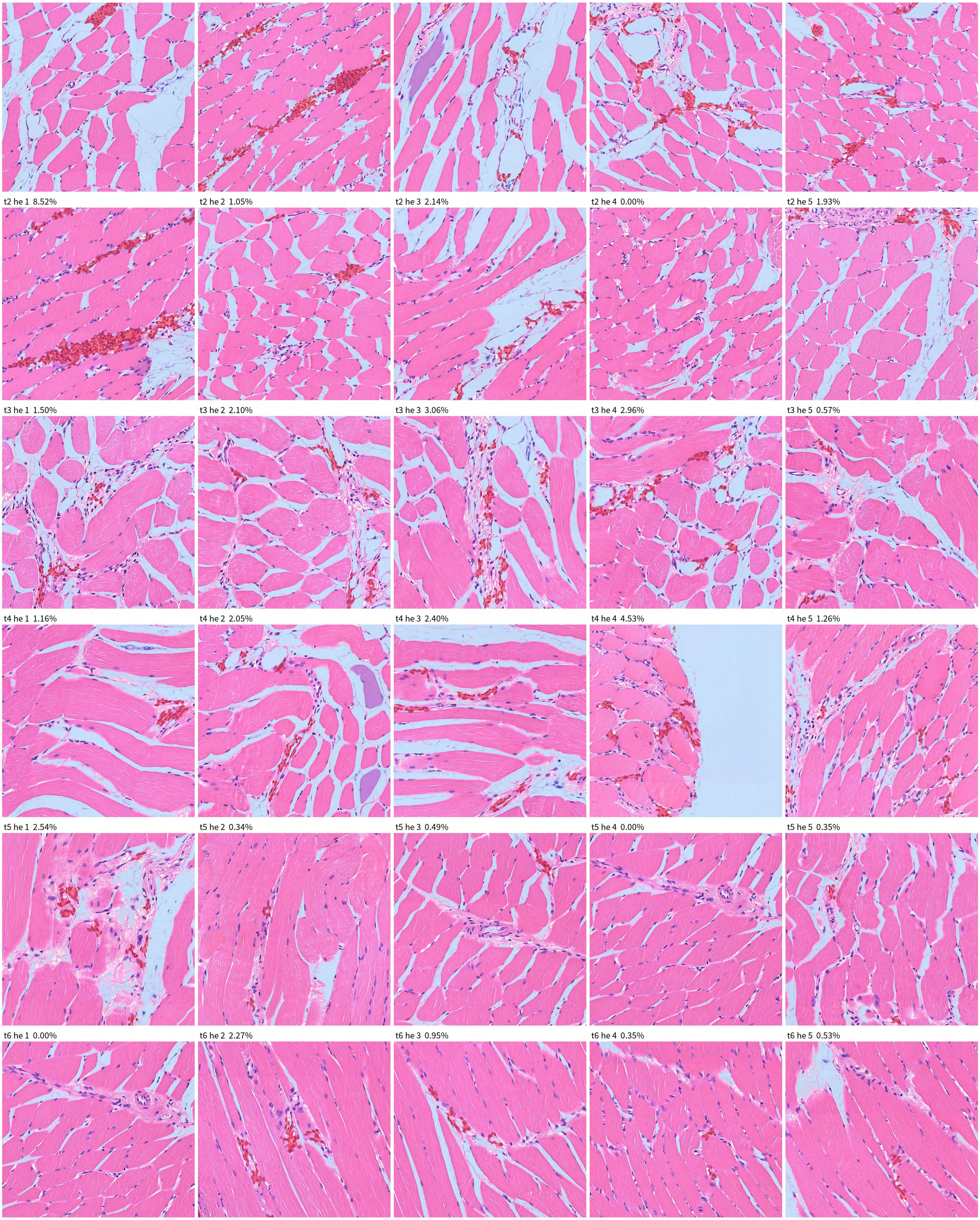

The same fixed analysis parameters were applied to all groups.

H&E inflammatory-infiltration analysis - MOD

Thirty fields (five fields per animal; six animals per group); labels show the quantified area percentage.

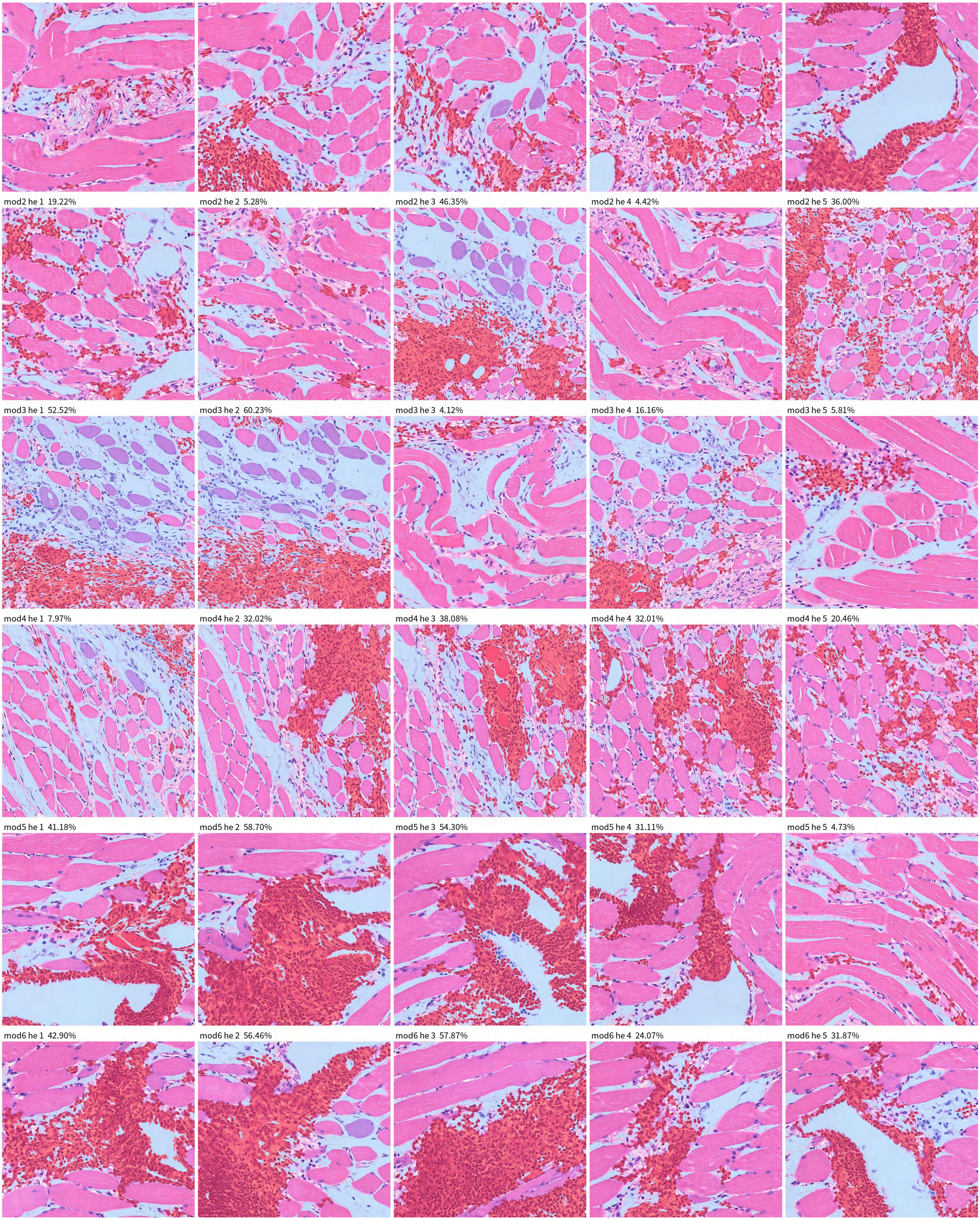

The same fixed analysis parameters were applied to all groups.

Masson fibrotic-area analysis - CON

Thirty fields (five fields per animal; six animals per group); labels show the quantified area percentage.

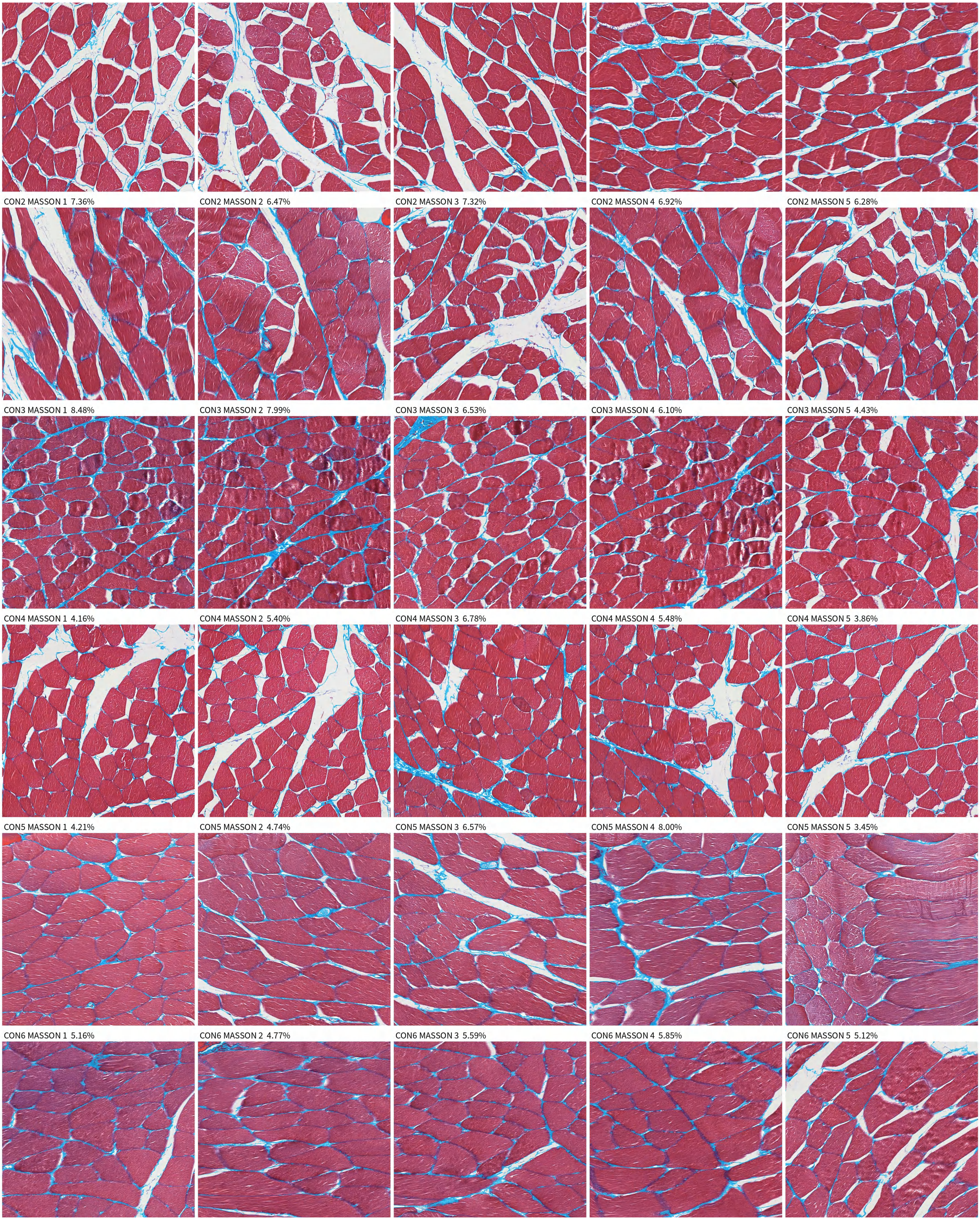

The same fixed analysis parameters were applied to all groups.

Masson fibrotic-area analysis - WAA-TENS

Thirty fields (five fields per animal; six animals per group); labels show the quantified area percentage.

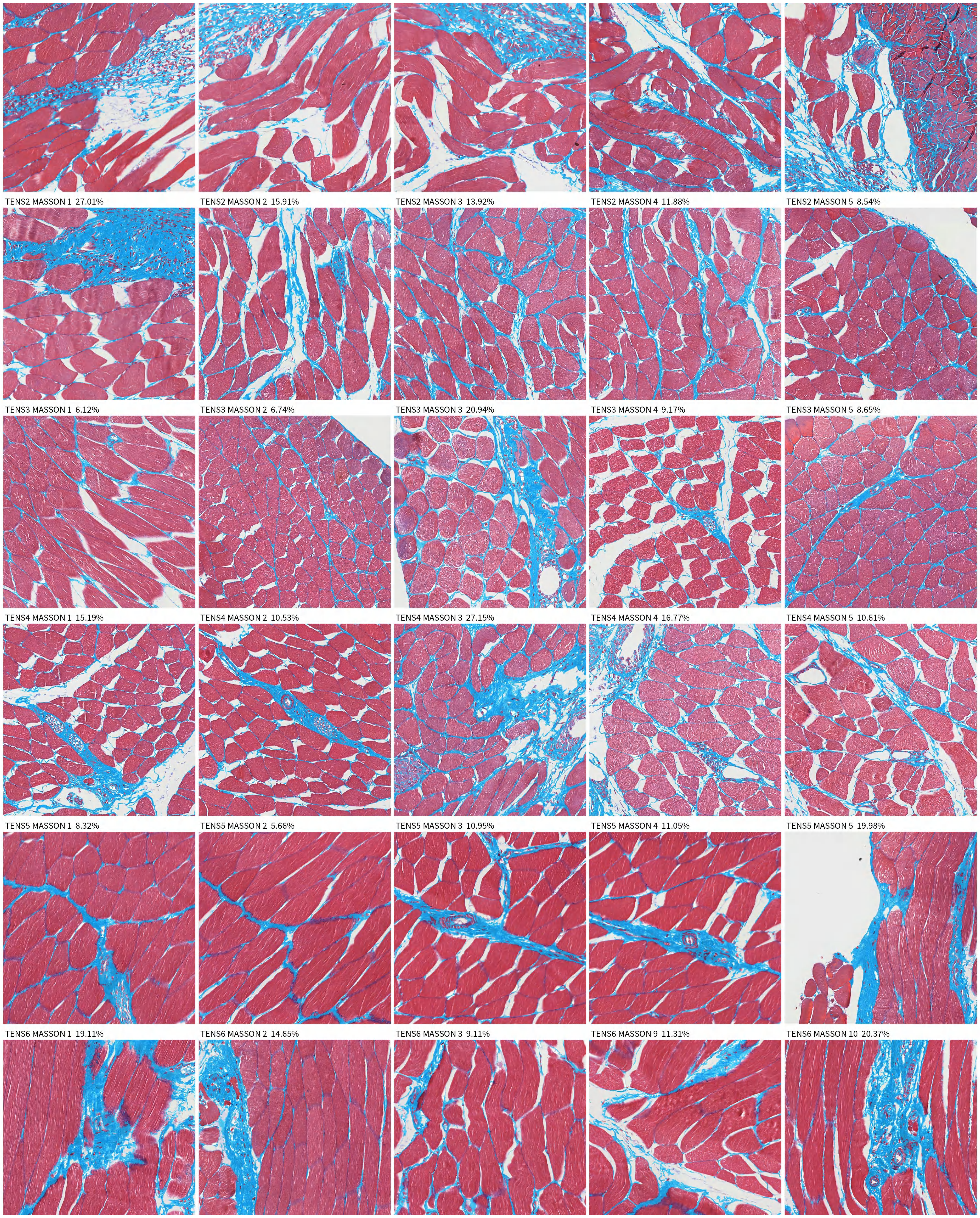

The same fixed analysis parameters were applied to all groups.

Masson fibrotic-area analysis - MOD

Thirty fields (five fields per animal; six animals per group); labels show the quantified area percentage.

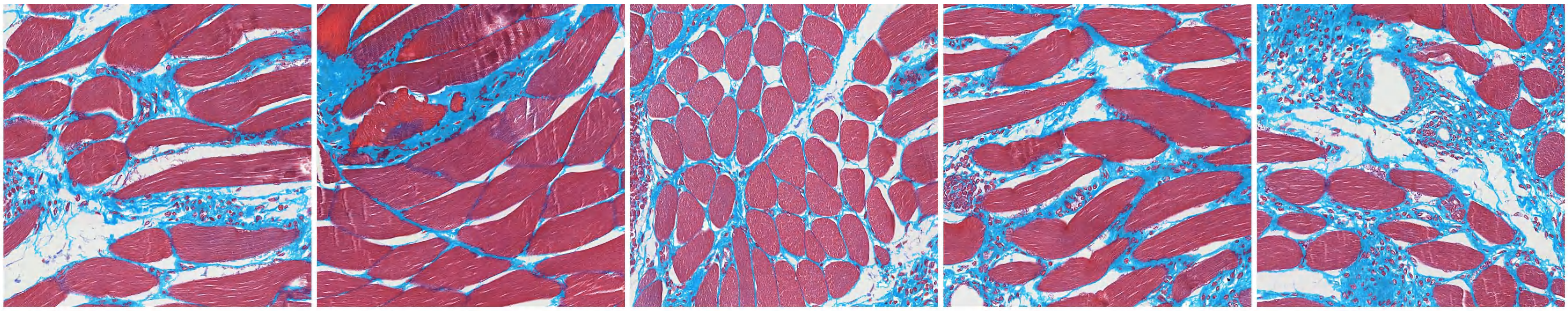

MOD2 MASSON 1 21.53% | MOD2 MASSON 2 44.15% | MOD2 MASSON 3 53.09% | MOD2 MASSON 4 40.15% | MOD2 MASSON 5 34.18%

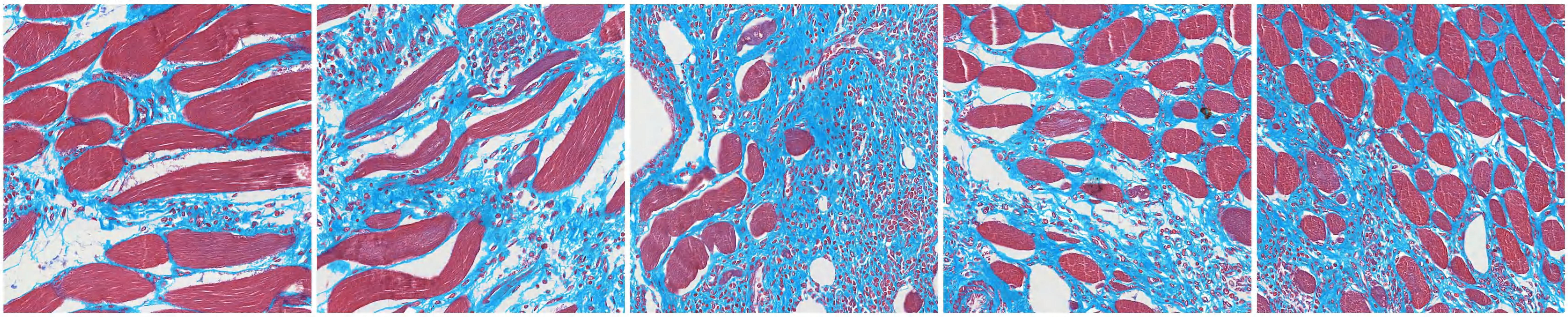

MOD3 MASSON 1 15.59% | MOD3 MASSON 2 42.26% | MOD3 MASSON 3 19.29% | MOD3 MASSON 4 43.95% | MOD3 MASSON 5 52.91%

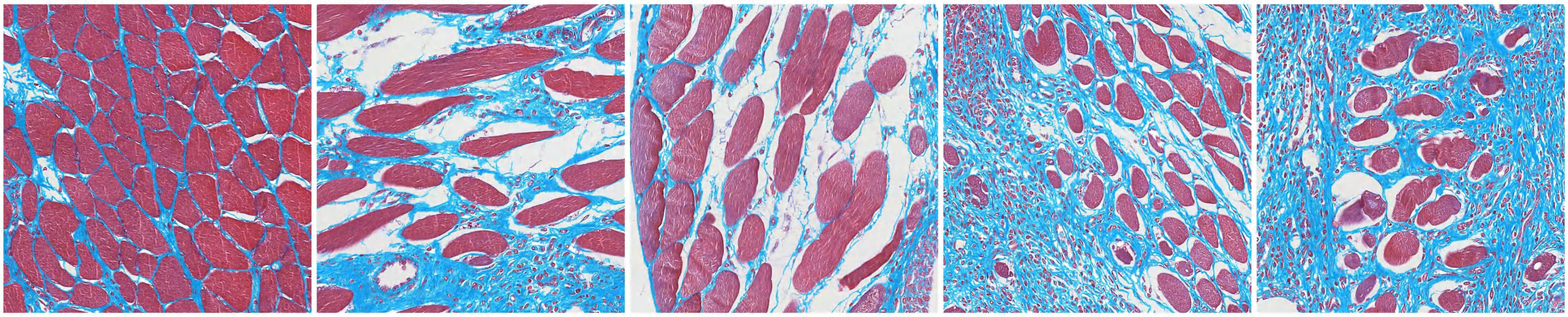

MOD4 MASSON 1 42.55% | MOD4 MASSON 2 45.74% | MOD4 MASSON 3 43.20% | MOD4 MASSON 4 46.29% | MOD4 MASSON 5 26.82%

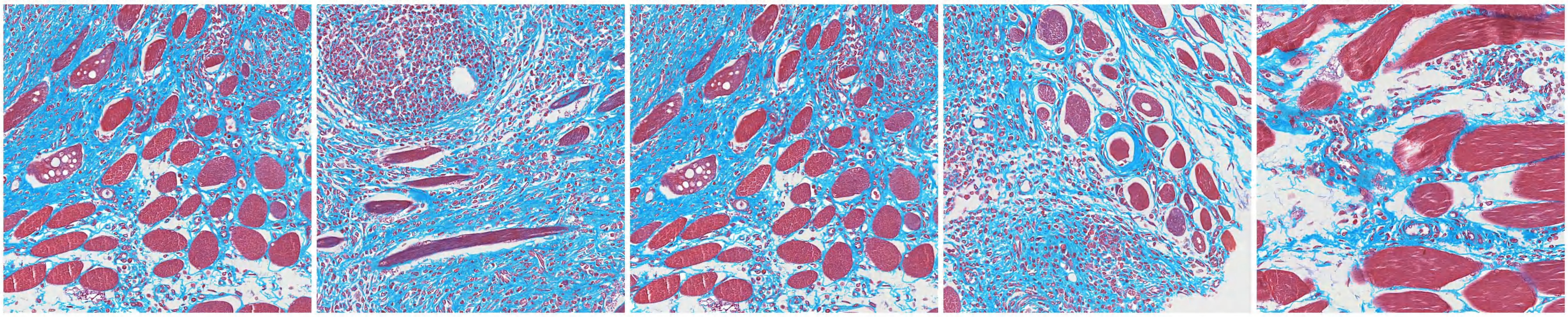

MOD5 MASSON 1 27.94% | MOD5 MASSON 2 23.98% | MOD5 MASSON 3 31.63% | MOD5 MASSON 4 37.18% | MOD5 MASSON 5 45.44%

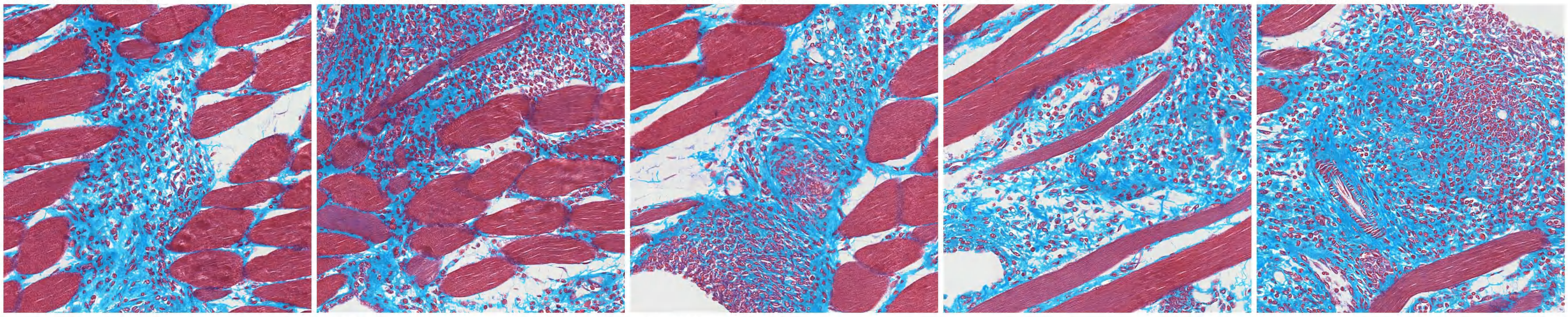

MOD6 MASSON 1 44.00% | MOD6 MASSON 2 38.98% | MOD6 MASSON 3 45.84% | MOD6 MASSON 4 15.12% | MOD6 MASSON 5 22.04%

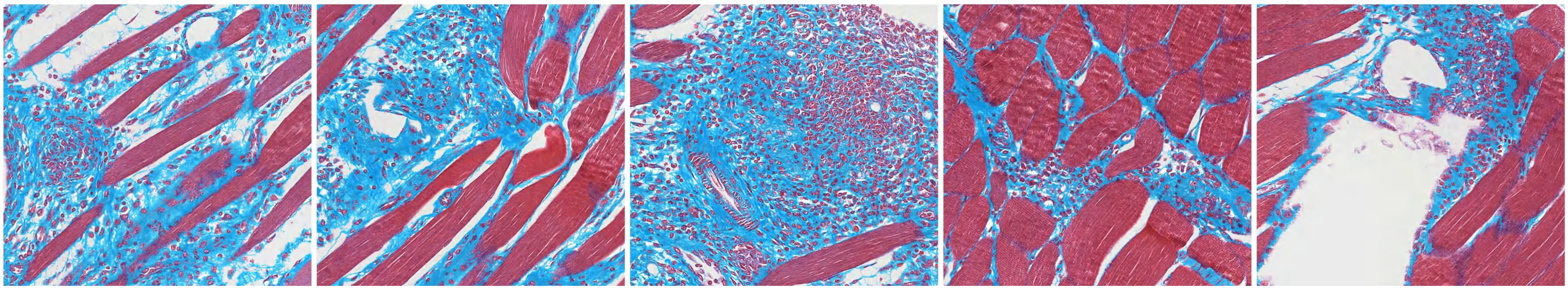

The same fixed analysis parameters were applied to all groups.

Minimum Feret diameter - CON

Final accepted myofiber profiles are outlined in green; one fixed parameter set was used for all groups.

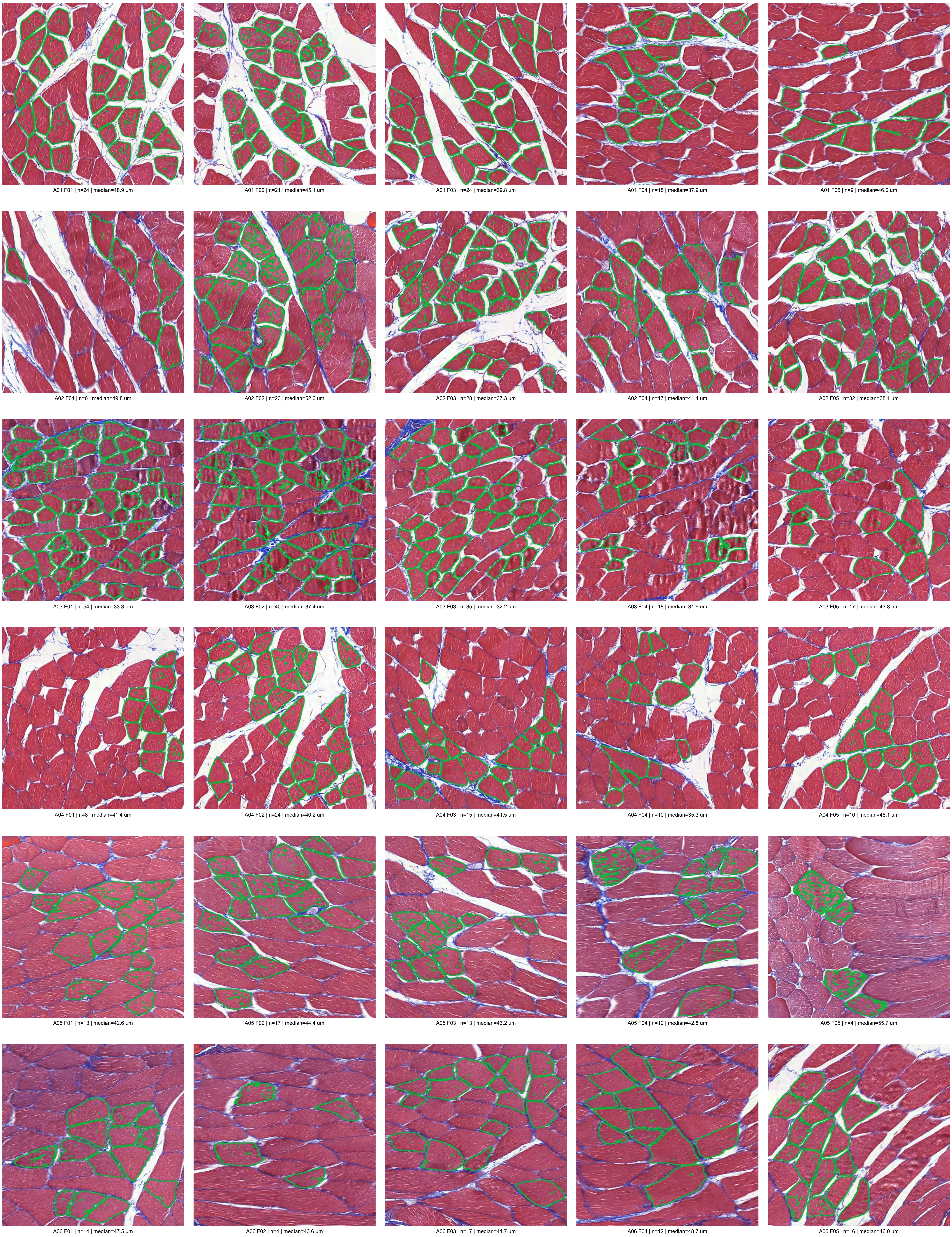

Minimum Feret diameter - WAA-TENS

Final accepted myofiber profiles are outlined in green; one fixed parameter set was used for all groups.

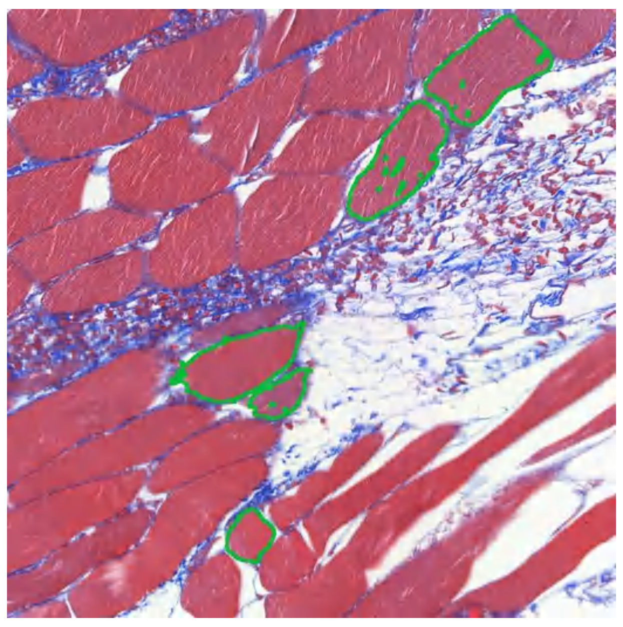

A01 F01 | n=5 | median=39.9 um

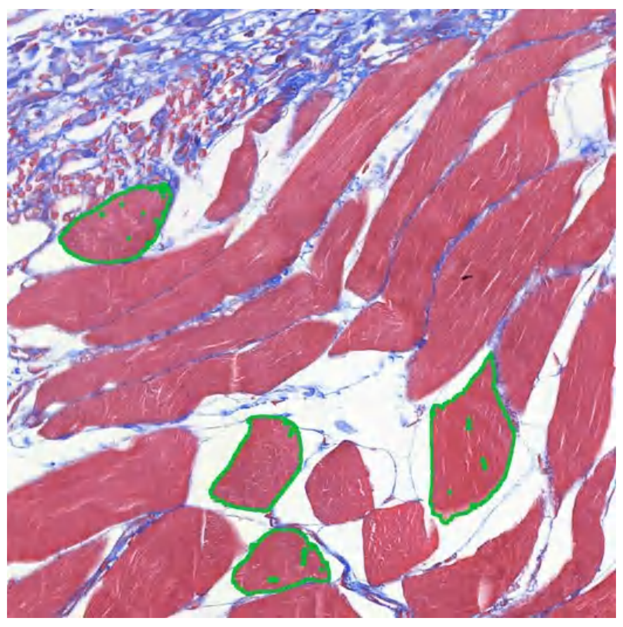

A01 F02 | n=4 | median=43.9 um

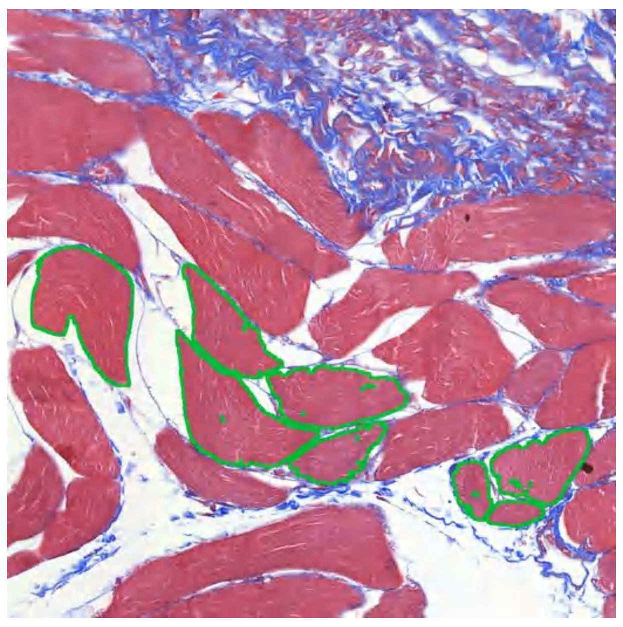

A01 F03 | n=8 | median=39.2 um

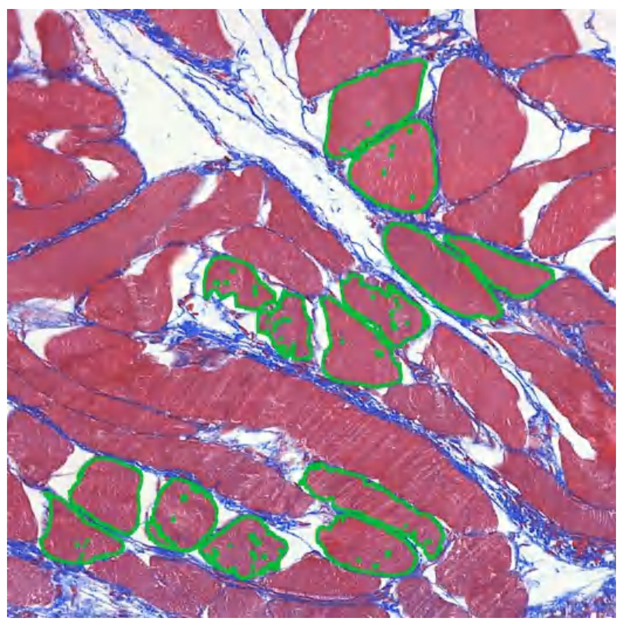

A01 F04 | n=12 | median=42.6 um

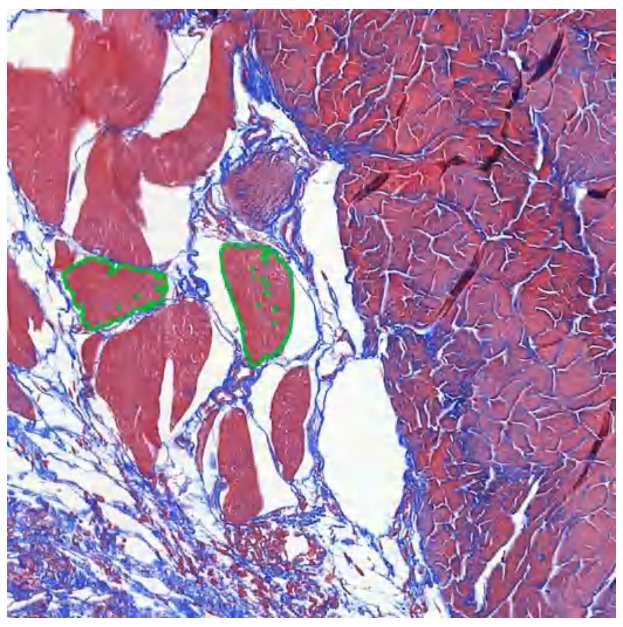

A01 F05 | n=2 | median=44.8 um

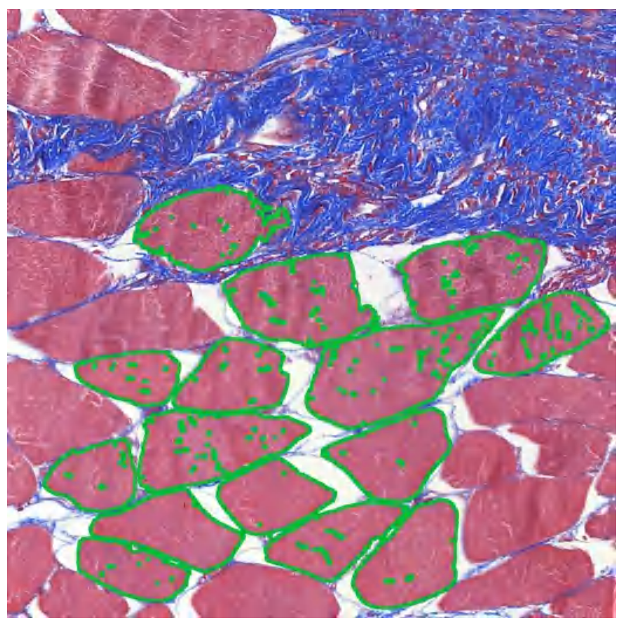

A02 F01 | n=12 | median=53.6 um

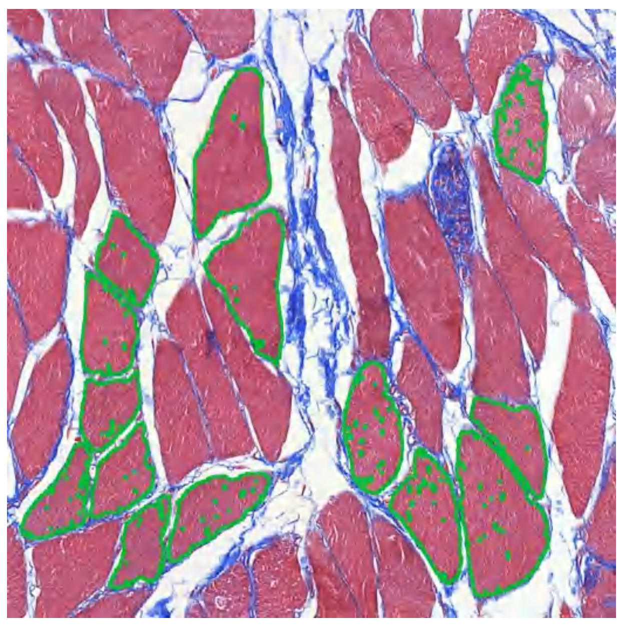

A02 F02 | n=14 | median=37.0 um

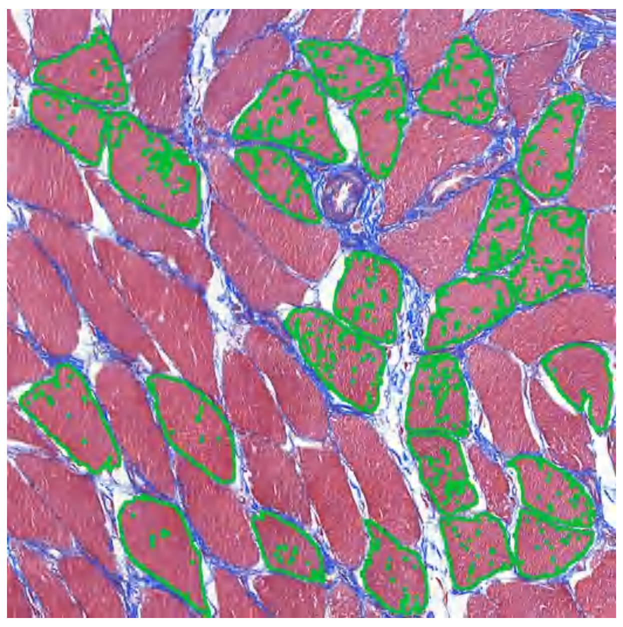

A02 F03 | n=25 | median=39.7 um

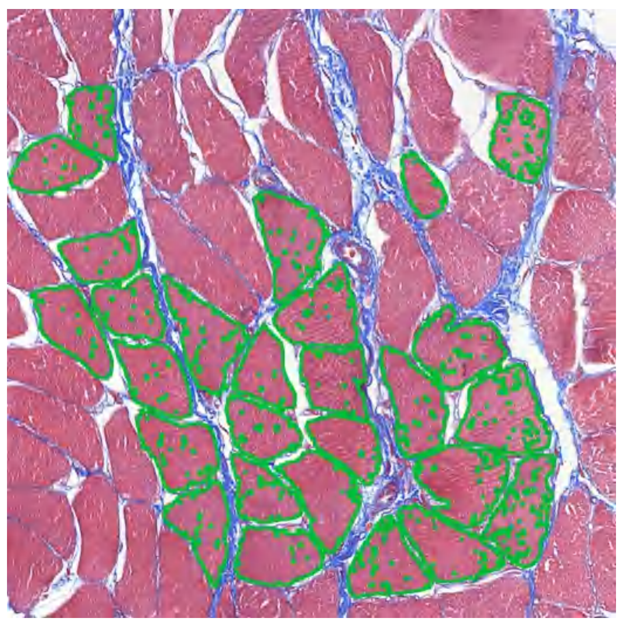

A02 F04 | n=26 | median=39.5 um

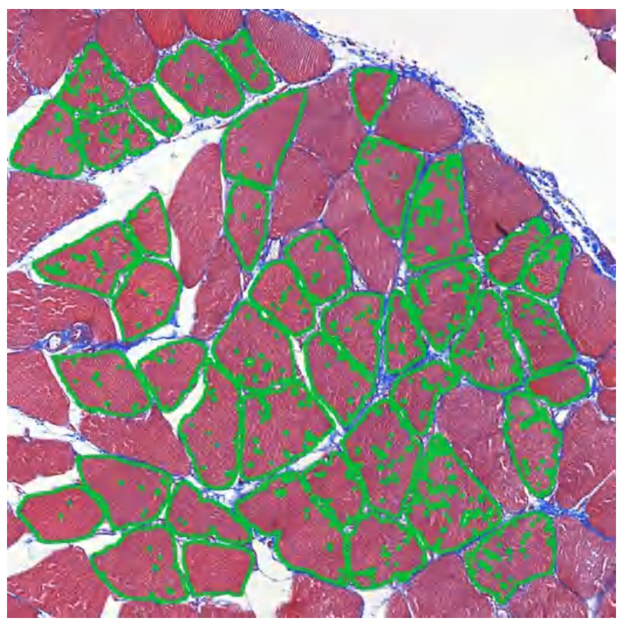

A02 F05 | n=42 | median=38.6 um

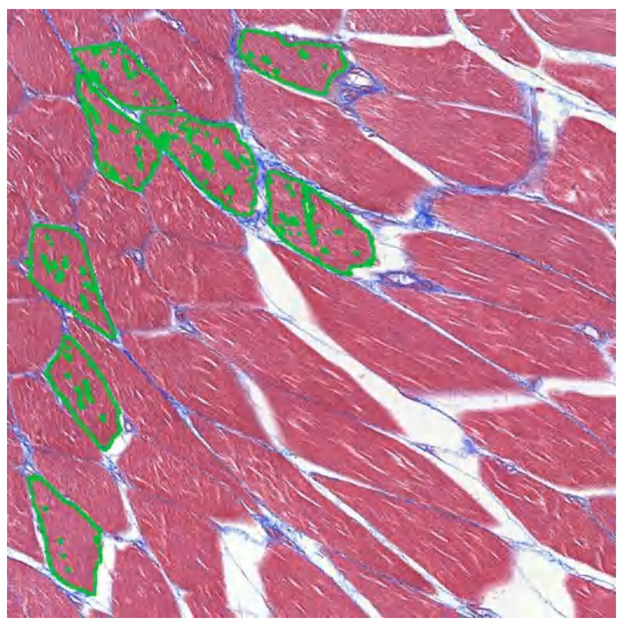

A03 F01 | n=8 | median=38.9 um

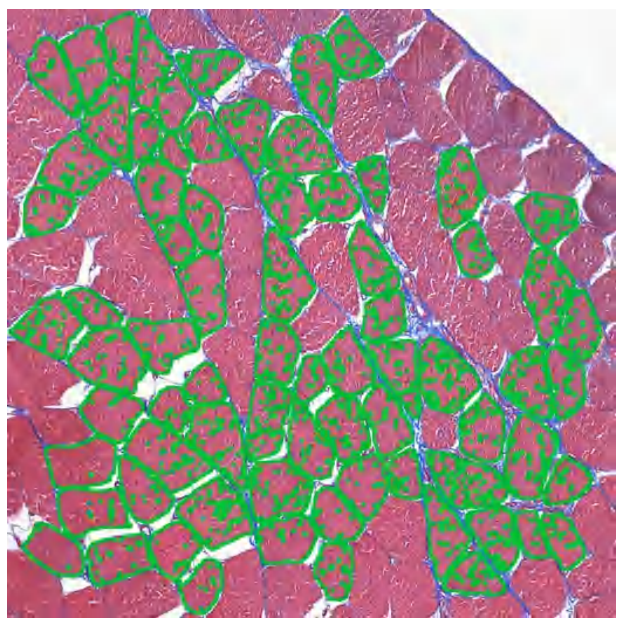

A03 F02 | n=71 | median=28.4 um

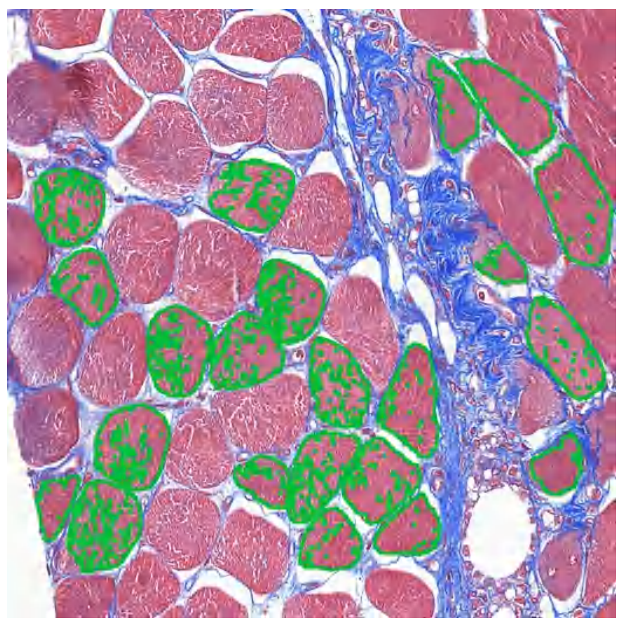

A03 F03 | n=22 | median=39.1 um

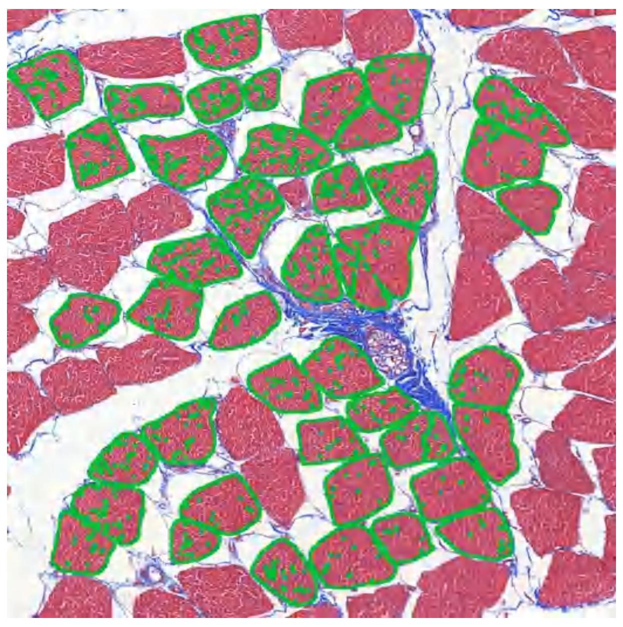

A03 F04 | n=34 | median=36.1 um

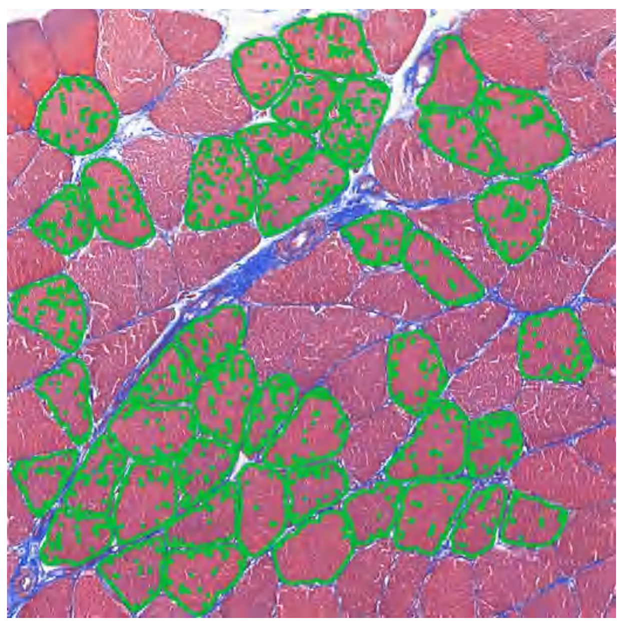

A03 F05 | n=43 | median=36.0 um

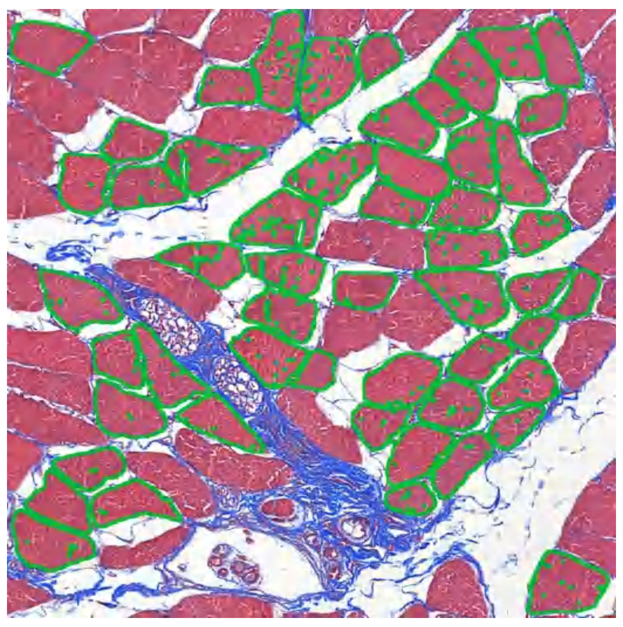

A04 F01 | n=51 | median=30.1 um

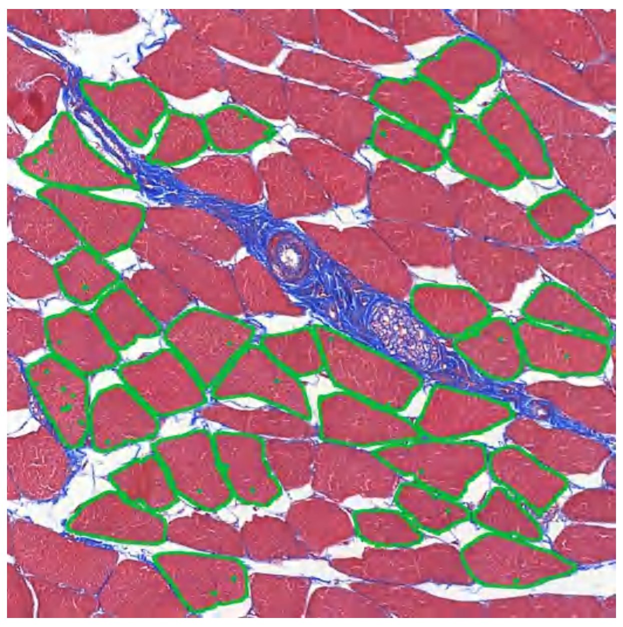

A04 F02 | n=33 | median=33.0 um

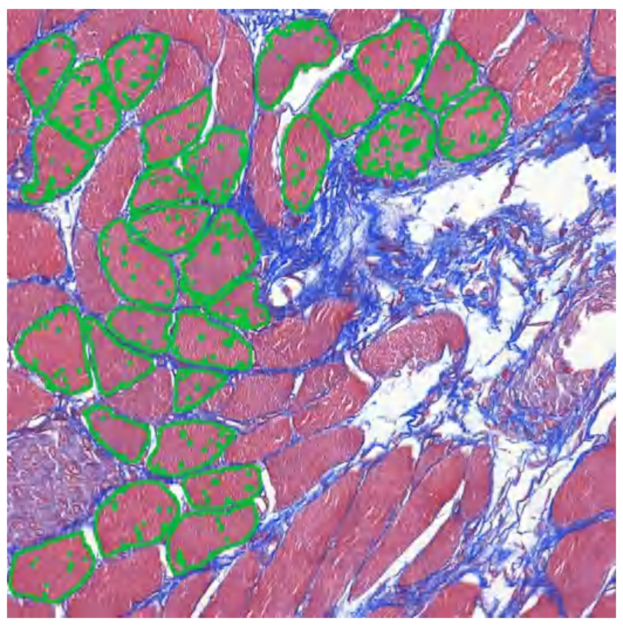

A04 F03 | n=29 | median=35.8 um

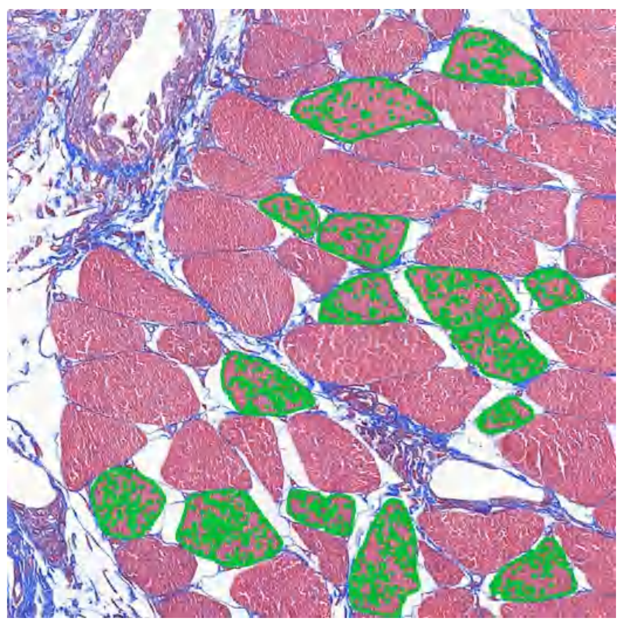

A04 F04 | n=15 | median=37.0 um

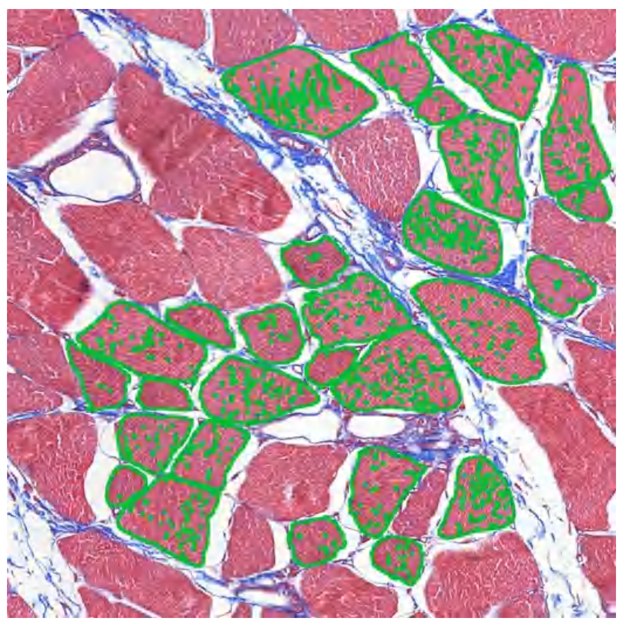

A04 F05 | n=27 | median=39.3 um

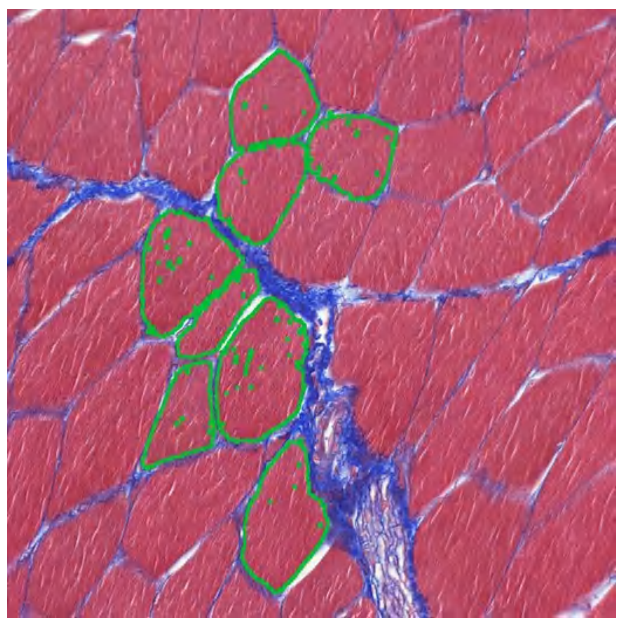

A05 F01 | n=6 | median=57.0 um

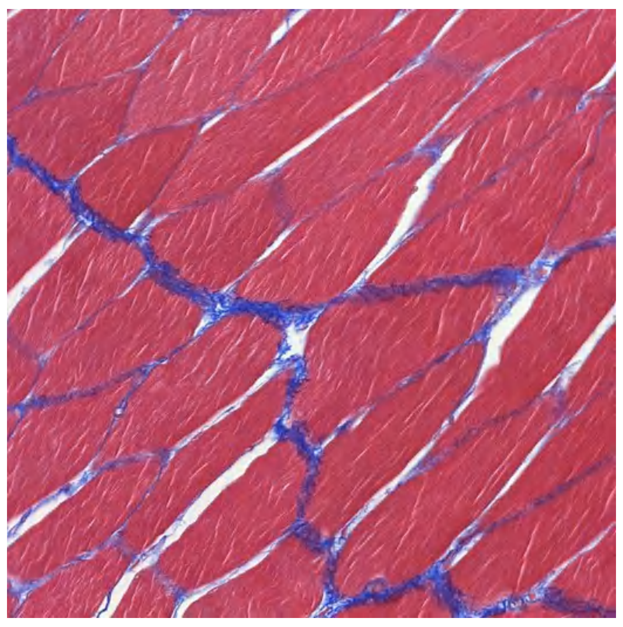

A05 F02 | n=0 | median=NA

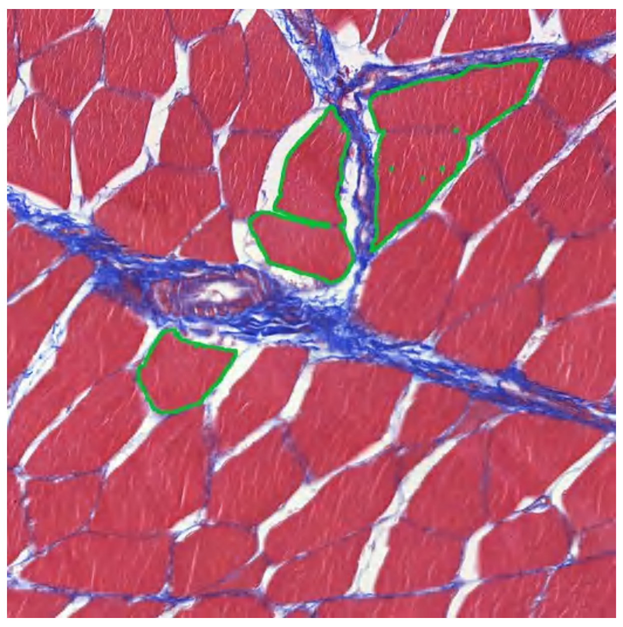

A05 F03 | n=3 | median=69.2 um

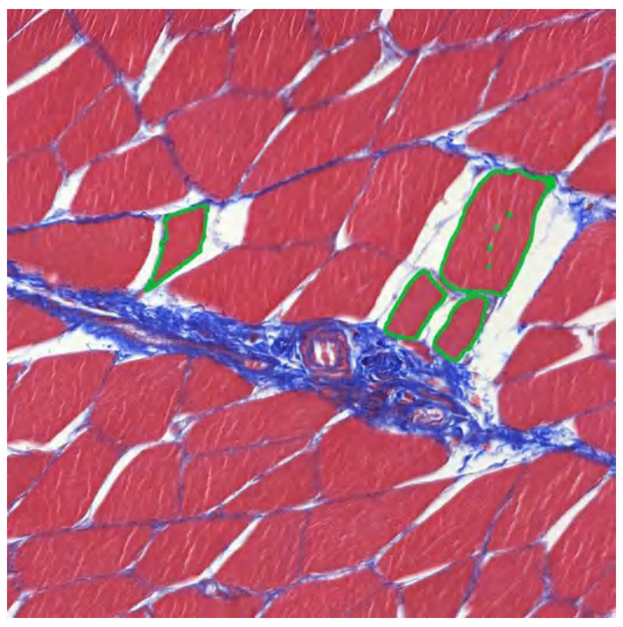

A05 F04 | n=4 | median=26.5 um

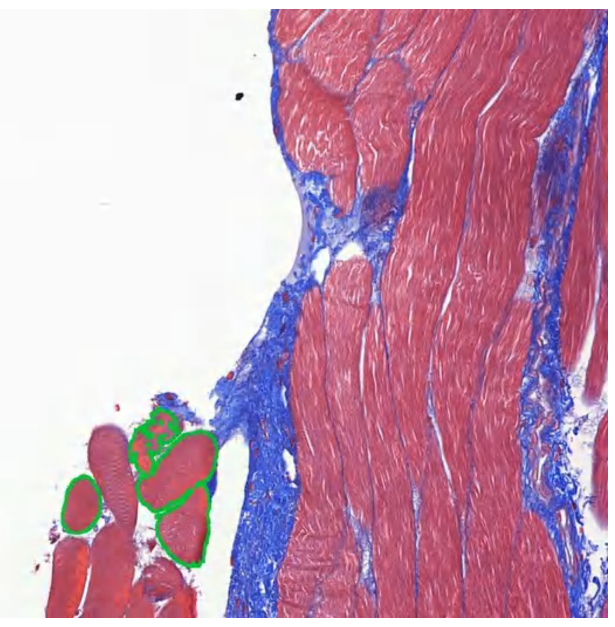

A05 F05 | n=2 | median=41.3 um

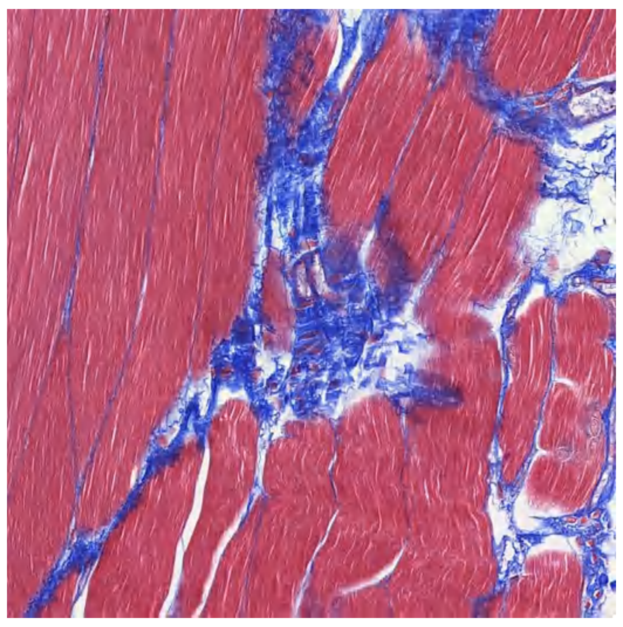

A06 F01 | n=0 | median=NA

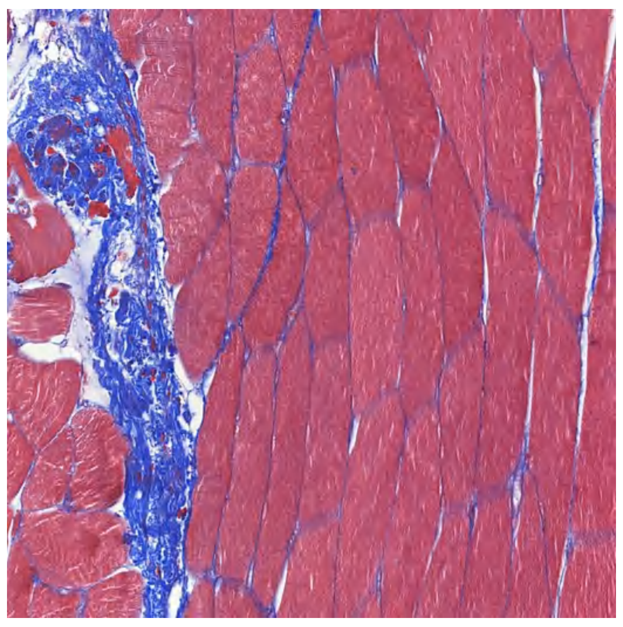

A06 F02 | n=0 | median=NA

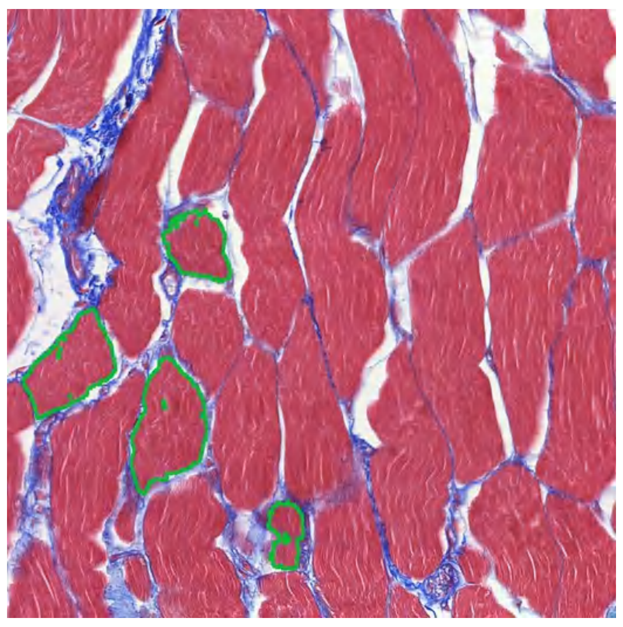

A06 F03 | n=4 | median=41.5 um

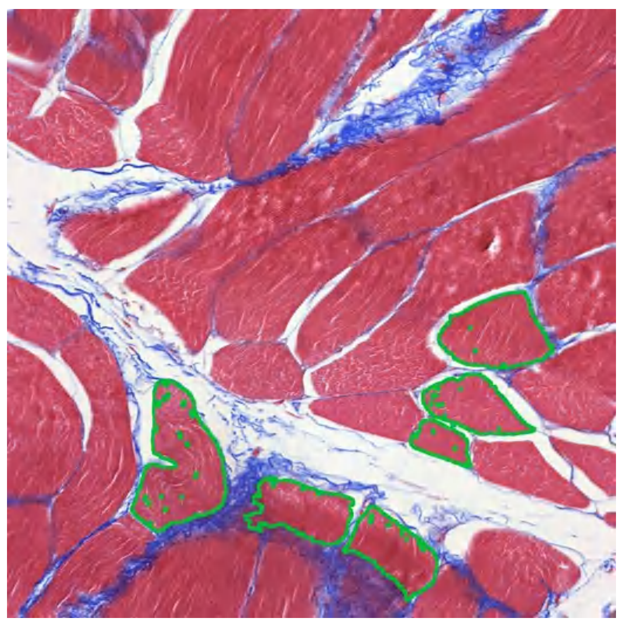

A06 F09 | n=6 | median=38.0 um

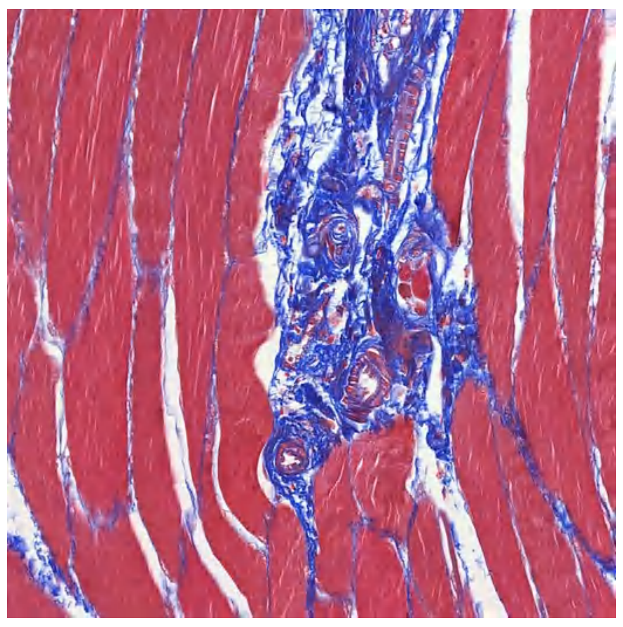

A06 F10 | n=0 | median=NA

Minimum Feret diameter - MOD

Final accepted myofiber profiles are outlined in green; one fixed parameter set was used for all groups.

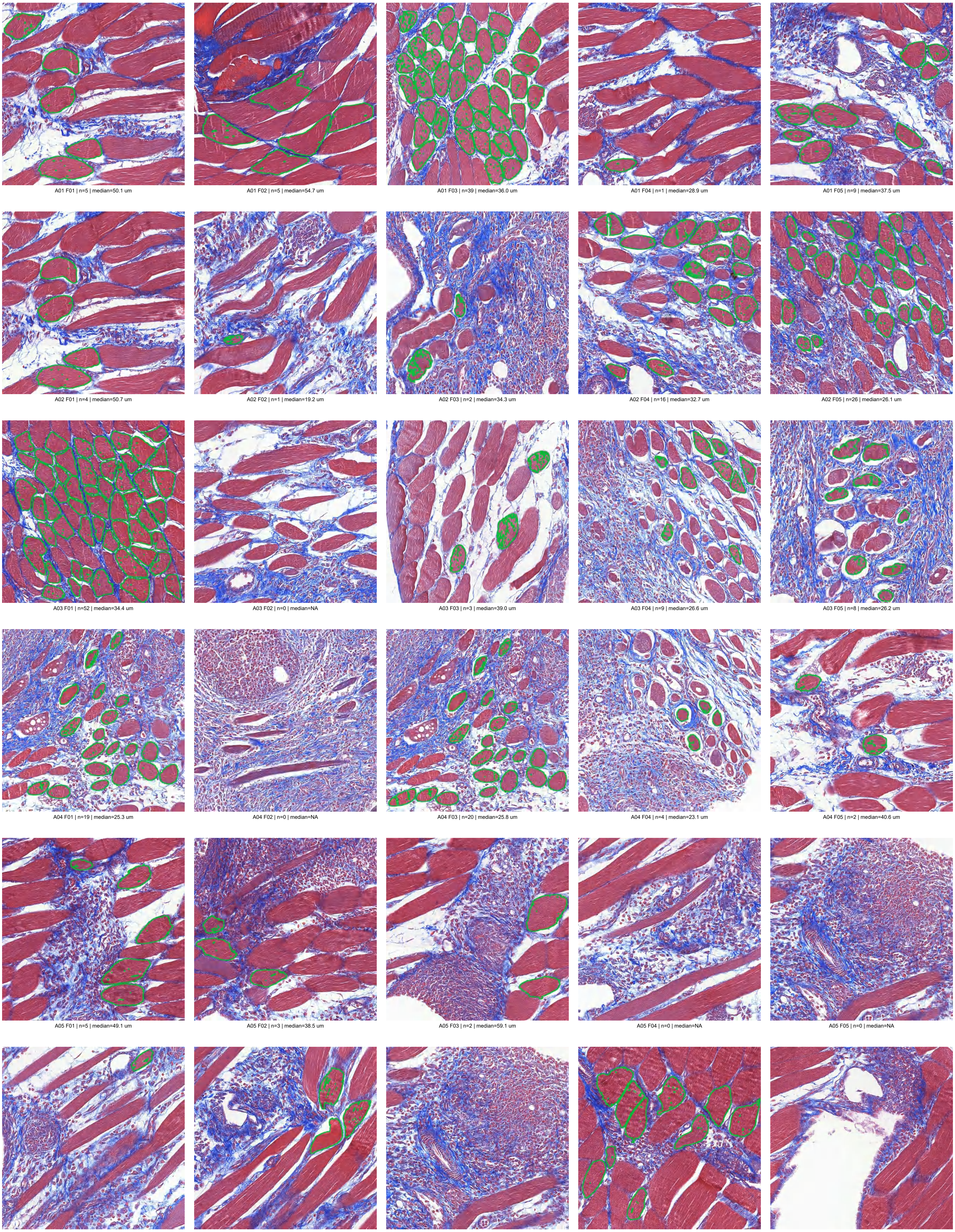

A01 F01 | n=5 | median=50.1 um
A01 F02 | n=5 | median=54.7 um
A01 F03 | n=39 | median=36.0 um
A01 F04 | n=1 | median=28.9 um
A01 F05 | n=9 | median=37.5 um

A02 F01 | n=4 | median=50.7 um
A02 F02 | n=1 | median=19.2 um
A02 F03 | n=2 | median=34.3 um
A02 F04 | n=16 | median=32.7 um
A02 F05 | n=26 | median=26.1 um

A03 F01 | n=52 | median=34.4 um
A03 F02 | n=0 | median=NA
A03 F03 | n=3 | median=39.0 um
A03 F04 | n=9 | median=26.6 um
A03 F05 | n=8 | median=26.2 um

A04 F01 | n=19 | median=25.3 um
A04 F02 | n=0 | median=NA
A04 F03 | n=20 | median=25.8 um
A04 F04 | n=4 | median=23.1 um
A04 F05 | n=2 | median=40.6 um

A05 F01 | n=5 | median=49.1 um
A05 F02 | n=3 | median=38.5 um
A05 F03 | n=2 | median=59.1 um
A05 F04 | n=0 | median=NA
A05 F05 | n=0 | median=NA

A06 F01 | n=1 | median=26.1 um
A06 F02 | n=3 | median=47.7 um
A06 F03 | n=0 | median=NA
A06 F04 | n=10 | median=43.3 um
A06 F05 | n=0 | median=NA
